# Sulfonium‐Stapled Peptides‐Based Neoantigen Delivery System for Personalized Tumor Immunotherapy and Prevention

**DOI:** 10.1002/advs.202307754

**Published:** 2024-04-11

**Authors:** Yaping Zhang, Leying Jiang, Siyong Huang, Chenshan Lian, Huiting Liang, Yun Xing, Jianbo Liu, Xiaojing Tian, Zhihong Liu, Rui Wang, Yuhao An, Fei Lu, Youdong Pan, Wei Han, Zigang Li, Feng Yin

**Affiliations:** ^1^ Pingshan Translational Medicine Center Shenzhen Bay Laboratory Shenzhen 518055 P. R. China; ^2^ State Key Laboratory of Chemical Oncogenomics, School of Chemical Biology and Biotechnology Peking University Shenzhen Graduate School Shenzhen 518055 P. R. China; ^3^ NeoCura Bio‐Medical Technology Co. Ltd. Shenzhen 518055 P. R. China

**Keywords:** nano‐carriers, neoantigens, personalized immunotherapy, stapling peptide, sulfonium

## Abstract

Neoantigen peptides hold great potential as vaccine candidates for tumor immunotherapy. However, due to the limitation of antigen cellular uptake and cross‐presentation, the progress with neoantigen peptide‐based vaccines has obviously lagged in clinical trials. Here, a stapling peptide‐based nano‐vaccine is developed, comprising a self‐assembly nanoparticle driven by the nucleic acid adjuvant‐antigen conjugate. This nano‐vaccine stimulates a strong tumor‐specific T cell response by activating antigen presentation and toll‐like receptor signaling pathways. By markedly improving the efficiency of antigen/adjuvant co‐delivery to the draining lymph nodes, the nano‐vaccine leads to 100% tumor prevention for up to 11 months and without tumor recurrence, heralding the generation of long‐term anti‐tumor memory. Moreover, the injection of nano‐vaccine with signal neoantigen eliminates the established MC‐38 tumor (a cell line of murine carcinoma of the colon without exogenous OVA protein expression) in 40% of the mice by inducing potent cytotoxic T lymphocyte infiltration in the tumor microenvironment without substantial systemic toxicity. These findings represent that stapling peptide‐based nano‐vaccine may serve as a facile, general, and safe strategy to stimulate a strong anti‐tumor immune response for the neoantigen peptide‐based personalized tumor immunotherapy.

## Graphical abstract

A sulfonium‐based stapling peptide carrier containing only nine amino acids is developed through computer‐aided screening for developing personalized nano‐vaccines. By cooperating with this carrier, a single neoantigen peptide could sufficiently trigger a robust and sustained tumor‐specific T cell response, further eliminating the established tumors in multiple tumor models by regulating the tumor infiltration microenvironment.

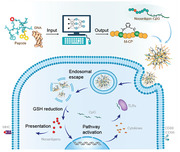

## 1 Introduction

The mRNA vaccine encoding 34 neoantigens in phase IIb clinical trial has ushered the main end in 2022, heralding the great promise of neoantigens as personalized vaccines for tumor treatment.^[^ ^1^ ^]^ Meanwhile, as the first approved personalized tumor vaccine for clinical trials, the progress of peptide‐based neoantigens has lagged behind the mRNA vaccines, which can be owed to the rapid metabolism, inefficient delivery to draining lymph nodes, and low immunogenicity of peptides.^[^ ^2^ ^]^ Even so, peptides still are the most direct and original forms for antigen presentation and subsequent T cell recognition,^[^ ^3^ ^]^ which are facile to manufacture, safe for clinical applications, and modifiable with various strategies.^[^ ^4^ ^]^ Given the facts above, peptide‐based neoantigen vaccines will be a new paradigm for personalized therapeutics by solving the fundamental limitation of neoantigen peptides related to cellular uptake and cross‐presentation processes. The combination of nanotechnology and immunotherapy provides a breakthrough to overcome these issues, and previous research has shown that nano‐carriers such as PEI or DNA origami containing multiple neoantigens can significantly improve the intensity of immune response induced by antigen peptides.^[^ ^5^ ^]^ While facing the problem of inadequate immune response to a single neoantigen peptide, it is imperative to develop safer, simpler, and more effective delivery carriers, which will be one of the shortcuts to promoting neoantigen‐based vaccines to clinical practice in the future.

**Figure 1:**
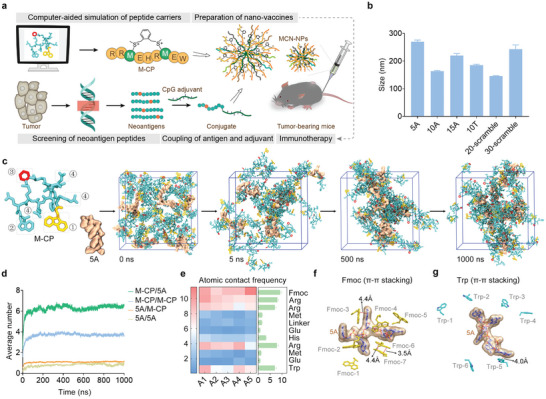
Characterization of assembly behaviors of the sulfonium‐based stapling peptide with nucleic acid. a) Schematic illustration of the nano‐vaccine preparation for personalized immunotherapy. First, by evaluating the interaction between nucleic acid and sulfonium‐based stapling peptide, computer‐aided simulation was used to redesign the candidate peptide carriers. On the other hand, the candidate neoantigen peptides derived from sequencing and prediction were conjugated with CpG adjuvant. Then, the negatively charged conjugate induced the self‐assembly with the positively charged stapling peptide into stable and safe nano‐vaccines. Once uptake by antigen presentation cells, the active sulfonium centers were reduced by the intracellular GSH, further inducing the disintegration of nano‐vaccine and the release of internal antigen and adjuvant, which could elicit a robust anti‐tumor response for tumor‐specific immunotherapy. b) Size analysis of nanoparticles assembled by M‐CP with different lengths of nucleic acids (N/P = 4) via dynamic light scattering. Nanoparticles showed similar particle sizes (150–250 nm) when N/P was 4. c) Molecular dynamics simulations of M‐CP and 5A in a 10 × 10 × 10 nm cubic box over 1000 ns. The gray dashed boxes represented the Fmoc group at the N‐end (①, yellow), tryptophan residue at the C‐end (②, light blue), benzene on the closed‐loop (③, red), and arginine (④, light blue), respectively. Bright blue represented the other amino acid residues of M‐CP. 5A was shown as a surface model. [5A]:[M‐CP] = 1:6. d) Intermolecular distribution of M‐CP and 5A inside the nanoparticles. M‐CP/5A showed the average number of M‐CP molecules around one 5A molecule. e) The heat map of the atomic contact frequencies between amino acid residues of M‐CP and each nucleotide of 5A, reflecting the strength of interaction forces. Higher frequencies corresponded to stronger interaction. The right bar chart represents the average interaction frequencies between a single amino acid residue and each nucleotide residue. π–π stacking between f) the Fmoc group / g) tryptophan of M‐CP and base planes of nucleic acid (5A). To simplify the structure, M‐CP was shown as 9‐fluorenyl (yellow) in (f) or β‐indolyl (light blue embedded with blue‐green N atom) in (g). 5A was shown as the perspective surface model. The red dash indicates the potential π–π stacking positions.

Here, we propose a general strategy utilizing a sulfonium‐based stapling peptide carrier with only nine amino acids to generate personalized nano‐vaccines, incorporating neoantigen peptides and nucleic acid‐based adjuvants for tumor therapy (**Figure** **1**a). For proof of concept, a single antigen was encapsulated in the carrier to prepare the nano‐vaccine. To achieve the co‐delivery and full encapsulation of neoantigens and adjuvants, reducible adjuvant‐neoantigen conjugates driven by sulfonium were prepared according to our previous strategy.^[^ ^6^ ^]^ We discovered that negatively charged CpG‐antigen conjugate （CpG, unmethylated cytosine‐guanine oligo‐deoxynucleotides） could rapidly induce the positively charged stapling peptide vectors to self‐assemble into regular and stable nano‐spheres in less than 30 min, due to the electrostatic interaction and π–π stacking. This reducible nano‐vaccine could contribute to changes in several biological phenomena, including promoting cytosolic delivery of antigens, prolonging the antigen presentation on antigen presentation cells (APCs), and stimulating the strong immunocytes activation in vitro and antigen‐specific anti‐tumor T cell response in vivo, which would significantly inhibit the tumor growth and lung metastasis of melanoma. Besides, it could also eliminate the established tumors, along with inducing potent cytotoxic T lymphocyte infiltration. Our study reveals that an efficient carrier, only combined with one single neoantigen, was sufficient enough to trigger a robust tumor‐specific T cell response. Considering the compatibility of this stapling peptide carrier with different CpG‐antigen conjugates, this attractive nanoplatform will also be suitable for embracing various neoantigens to avoid immune escape and might provide a new avenue for the design of a universal tumor vaccine with “shared” neoantigens in the future.^[^ ^7^ ^]^

## 2 Results and Discussion

### 2.1 Molecular Dynamics Simulate the Assembly Characteristics of Sulfonium‐Based Stapling Peptide

Neoantigens in coordination with adjuvants are essential to trigger a robust tumor‐specific immune response.^[^ ^8^ ^]^ As one of the approved adjuvants with only a single component, unmethylated CpG motifs were used here to couple with neoantigen peptide based on a reversible sulfonium center, to ease the co‐delivery and improve the immunogenicity of neoantigens.^[^ ^2^, ^9^ ^]^ However, the negatively charged CpG‐neoantigen conjugate would face the same membrane penetration problem as the siRNA drugs and mRNA vaccines,^[^ ^10^ ^]^ which poses another major challenge for neoantigen peptide‐based immunotherapy. Inspired by our recent study on the nucleic acid‐induced peptide vectors self‐assembly strategy,^[^ ^11^ ^]^ which can efficiently deliver siRNA into tumor cells in vivo to silence target gene, thus, we extended the stapling peptide nano‐carriers to the delivery of CpG‐neoantigen conjugates (Figures S1 and S2, Supporting Information). Firstly, we performed a computer‐aided simulation to evaluate the groups that play an important role in the stapling peptide vectors and provide guidance for the selection of the CpG‐neoantigen conjugate peptide carriers.

Limited by the current assembly simulation technology, 5A, a simple representative of nucleic acid‐type cargo, was put into a 10 × 10 × 10 nm cubic box for the similar assembly behavior of 5–40 nt single‐stranded DNAs at N/P = 4 (Figure 1b, and Figure S3a, Supporting Information). After inserting slightly excessive M‐CP (methionine‐closed peptide) carriers (N/P = 4.8, corresponding [M‐CP]: [CpG‐OVA] = 6:1), 5A and M‐CP were co‐assembled into large clusters with an average of one 5A molecule surrounded by 6–7 M‐CP molecules in 500 ns (Figure 1c,d, and Figure S3b, Supporting Information). We found that the Fmoc group, tryptophan, and three arginines on M‐CP mainly participated in the interaction with 5A (Figure 1e). Further analysis showed that the Fmoc group at the N‐end and tryptophan at the C‐end of M‐CP stabilized 5A through π–π staking (Figure 1f,g). In addition, with the stapling of M‐CP, the original three independent arginines on M‐CP were fixed into a “claw‐like” structure and interacted with the phosphoric acid skeleton of 5A to the maximum extent via hydrogen bonding interaction (Figure S3c, Supporting Information). Once the loop was opened, the atomic forces between 5A and the peptide carriers (especially Fmoc, tryptophan, and arginine) were significantly decreased (Figure S3d,e, Supporting Information), along with a 34% decrease in the encapsulation rate (Figure S3f, Supporting Information). Note that, these molecular interactions were significantly different from the traditional cationic peptide carriers (TAT or R8, containing over 8 basic amino acids^[^ ^12^ ^]^), as the total positive charges of M‐CP were four (six positive charges were provided from three arginines, one histidine, and two positive sulfonium centers, and two glutamates provided two negative charges, Figure S1, Supporting Information). These simulation results revealed that the conformation fixation of linear peptide played an important role in carrier assembly, and showed the outstanding advantage of sulfonium‐based stapling peptide carriers for the nucleic acid‐type cargo (like CpG and CpG‐neoantigen conjugate) delivery.

According to the simulation results, we designed a mini M‐CP‐like peptide library by adjusting the key groups on M‐CP (Figure S3g, Supporting Information) and further evaluated its encapsulation performance with CpG adjuvant through wet‐lab tests. When the molar ratio raised more than 150 ([M‐CP]: [CpG]), the CpG substrate gradually disappeared in gel electrophoresis and assembled with M‐CP into 67 nm nanospheres (Figure S3g,h, Supporting Information). However, Ac‐M‐CP (without the Fmoc end) could hardly be assembled with CpG adjuvant, even if the molar ratio was higher than 300 ([Ac‐M‐CP]:[CpG], Figure S3i, Supporting Information). Once the side chain loop was opened (M‐LP), the encapsulation effect of peptide carriers with CpG was significantly reduced (Figure S3i, Supporting Information). The same phenomenon was also observed in the M‐LP‐S^+^ group (Figure S3j, Supporting Information), which was consistent with the lower encapsulation rate in the computer‐aided simulation, indicating the important role of the peptide conformational fixation on co‐assembly efficiency. Moreover, compared with M‐CP‐S’, M‐CP with a larger hydrophobic side chain showed more conducive to assembly (Figure S3j, Supporting Information), which was supported by the previous report.^[^ ^13^ ^]^ In short, multiple factors could affect the assembly behaviors of peptides, and we speculated that this sulfonium‐based peptide (M‐CP) with only nine amino acids will be a suitable candidate carrier for the delivery of nucleic acid‐type cargos like CpG adjuvant and CpG‐neoantigen conjugates.

### 2.2 Sulfonium‐Based Stapling Peptide for Antigens and Adjuvants Co‐Delivery

**Figure 2:**
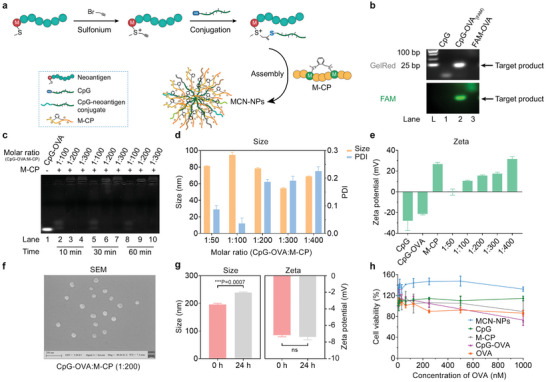
Design and characterization of nanoparticles for neoantigen and CpG adjuvant co‐delivery. a) Schematic illustration of the preparation of sulfonium‐based stapling peptide‐based nano‐vaccines (MCN‐NPs). The propargyl sulfonium at the methionine of neoantigen specifically reacted with sulfhydryl‐modified CpG adjuvant to increase the electronegativity of the antigen. Then, the negatively charged CpG‐neoantigen conjugate rapidly induced the self‐assembly with the positively charged stapling peptide (M‐CP) into uniform nano‐spheres. b) Gel electrophoresis (10% PAGE) analysis of CpG‐OVA conjugate. c) Gel electrophoresis (3% agarose) analysis of substrate ratio and time on the assembly of nanoparticles. d) Dynamic light scattering analysis on particle size and e) zeta potential of MCN‐NPs with different substrate ratios. f) Scanning electron microscope image of MCN‐NPs ([CpG‐OVA]:[M‐CP] = 1:200). Scale bar = 200 nm. g) The change of particle size and zeta potential of MCN‐NPs after 24 h incubation with 10% serum. h) Cytotoxicity of different nanoparticle formulations (calculated based on OVA concentration) in RAW264.7 cells. [CpG‐OVA]:[M‐CP] = 1:300. The data were presented as mean ± SEM (*n* = 3) from three independent experiments. Before conducting a significant difference analysis, the normality of data was analyzed by the “normality and lognormality Tests” option. Then, data in 2g were analyzed by parametric *t*‐test with two‐tailed *p*‐value (*p < 0.05, **p < 0.01, ***p < 0.001, ****p < 0.0001).

For the co‐delivery of antigen and adjuvant, negatively charged CpG‐neoantigen conjugates were prepared, followed by introducing the positively charged M‐CP for self‐assembly (**Figure** **2**a). Here, the OVA_257‐264_ peptide was first used as the model antigen for proof of concept. To strictly regulate the ratio and achieve the intracellular release of antigens and adjuvants via GSH‐reduction, we synthesized restorable CpG‐OVA conjugates (Figure 2b, and Figures S4 and S5, Supporting Information) by coupling propargyl sulfonium‐modified OVA peptide to sulfhydryl‐modified CpG as described in our previous reports.^[^ ^6^ ^]^ When incubated with M‐CP at room temperature for 10 min under various buffers (Figure 2c, and Figure S6a,b, Supporting Information), CpG‐OVA was almost completely encapsulated into nanoparticles (named MCN‐NPs) with molar ratios higher than 200 ([M‐CP]:[CpG‐OVA]). Especially when the ratios were 200 and 300 (Figure 2d–f), nanoparticles were uniformly dispersed with a diameter of about 70 nm, polydispersity indexes were less than 0.2, zeta potential ranged from +15 to +20 mV, and the onset aggregation temperatures exceeded 50 °C (Figure S6c, Supporting Information). By comparing the microscopic parameters of nanoparticles with CpG or CpG‐OVA conjugate cargos, we found that the hydrophobic OVA peptide at the end of CpG might promote the stability of nano‐vaccines, manifested by the higher *T*_m_ and *T*_agg_ values of MCN‐NPs compared with M‐CP‐CpG (Figure S6d,e, Supporting Information). These results were consistent with the previous molecular dynamics simulation where the hydrophobic groups were crucial for assembly (Figure 1).

We next evaluated the stability and safety of MCN‐NPs. Importantly, these ready‐to‐use nano‐vaccines (MCN‐NPs) were stable at 50 °C under the heating program of 25–95 °C (Figure S6c,e, Supporting Information) and could be stored at 4 °C for at least 12 weeks without significant changes in particle size and zeta potential (Figure S6f,g, Supporting Information). Even under serum, simulated gastrointestinal buffer, and DNase buffer, MCN‐NPs still maintained stable for more than 24 h (Figure 2g; Figure S6h, Supporting Information). Specifically, the negative zeta potential and particle size change of MCN‐NPs might be due to the adsorption of negative ions, but it does not affect the subsequent cellular uptake of nanoparticles (Figure 2g). Besides, once exposed to a simulated intracellular environment containing GSH, the MCN‐NPs could gradually disassemble through the reduction of sulfonium centers on M‐CP, further releasing the internal CpG‐OVA conjugate (Figure S7a,b, Supporting Information). In contrast, CCN‐NPs (without sulfonium centers) could hardly release the cargo even under 10 mM GSH buffer (Figure S7c–e, Supporting Information). These results demonstrated that the sulfonium‐based nano‐vaccines could be stably stored in vitro and release internal antigens and adjuvants triggered by intracellular GSH. In addition, neither M‐CP peptide nor MCN‐NPs could inhibit cells (RAW264.7, HeLa, and 293T cells) proliferation or cause hemolysis in blood cells even at a concentration of 300 µM (Figure 2h; Figure S8, Supporting Information), showing the excellent bio‐safety of this peptide nano‐carrier at the cellular level.

### 2.3 MCN‐NPs Induce Sustained Antigen Presentation and Enhanced APCs Activation

**Figure 3:**
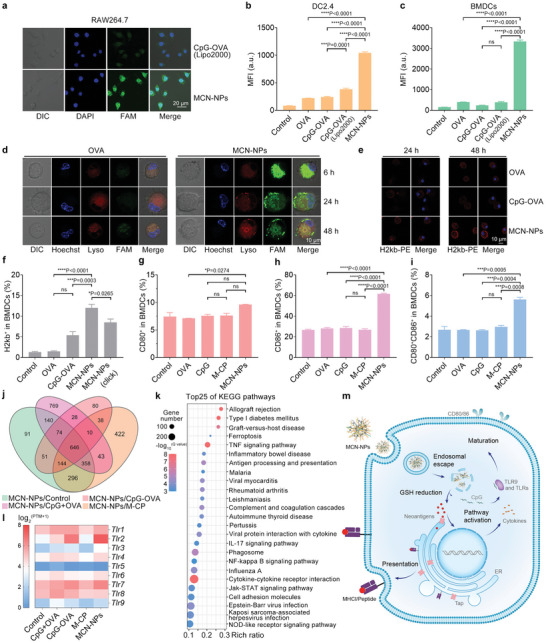
MCN‐NPs stimulate durable antigen presentation and strong immune cell activation. a) Localization of internalized OVA peptide in RAW264.7 cells after 4 h incubation with CpG‐OVA (Lipo2000) or MCN‐NPs. OVA was used as a model neoantigen in this experiment. A commercialized lipofectamine 2000 aided transfection group (CpG‐OVA (Lipo2000)) was used as the positive control. OVA peptide was labeled by FAM (green). DAPI (blue) stained the cell nuclei. Scale bar = 20 µm. The mean fluorescence intensity (MFI) of b) DC2.4 cells and c) BMDCs measured by flow cytometry, following 4 h incubation with MCN‐NPs. d) BMDCs were treated with free OVA peptide or MCN‐NPs for 6, 24, and 48 h. Hoechst (blue) was used to stain the cell nucleus. FAM (green) was employed to label the OVA peptide. Lysotracker (Lyso, red) was used as the lysosome dye. Scale bar = 10 µm. e) Confocal laser scanning microscopy (CLSM) or f) flow cytometry analysis of BMDCs with staining of the OVA‐MHC complex by PE‐labeled 25‐D1.16 H2kb antibody at 24 or 48 h. DAPI (blue) stained cell nuclei. Scale bar = 10 µm. g–i) Maturation of BMDCs after MCN‐NPs treatment for 24 h determined by the percentage of CD80^+^, CD86^+^, and CD80^+^CD86^+^cells. j) Venn/UpsetR figure of differential genes of MCN‐NPs treatment group versus control, CpG and OVA physical mixing group (CpG+OVA), CpG‐OVA conjugate group, or M‐CP peptide group in peritoneal macrophages. k) Top 25 of KEGG enrichment pathways with 646 differential genes in (j). l) Clustering thermogram of differential genes for the toll‐like receptor‐mediated pathways after MCN‐NPs treatment. m) Schematic illustration showing the mechanism of the cellular uptake and lysosomal escape of MCN‐NPs, leading to enhanced antigen presentation and further activation of the downstream immune pathways. The data were presented as mean ± SEM (*n* = 3) from three independent experiments. Data were analyzed by one‐way ANOVA with Turkey multiple comparisons post‐test (*p < 0.05, **p < 0.01, ***p < 0.001, ****p < 0.0001).

Next, the cellular uptake behaviors and localization of MCN‐NPs in immunocytes were evaluated by confocal laser scanning microscopy and flow cytometry. We observed that the free OVA peptide could hardly penetrate the cell membrane (Figure S9, Supporting Information). Whereas, RAW264.7 cells incubated with MCN‐NPs for 4 h displayed a stronger FAM signal than the commercialized transfection reagent‐assisted treatment group (CpG‐OVA(Lipo2000)) (**Figures** **3**a, and S9a, Supporting Information). Similarly, these phenomena were found in DC2.4 cells and the bone marrow‐derived dendritic cells (BMDCs, immature DCs) (Figures 3b,c, and S9b,c,e,f, Supporting Information). Meanwhile, the partial co‐localization of the intense green fluorescence with lysosomes in the MCN‐NPs treatment group was also observed at 48 h, which was still higher than the free OVA peptide treatment group (Figures 3d, and S9g,i,j, Supporting Information), indicating the effective delivery of antigens by M‐CP nano‐carrier. After the internalization of nanoparticles by APCs, the release and presentation of internal antigens were crucial for subsequent T cell activation.^[^ ^14^ ^]^ According to the confocal microscopy images, we discovered that with the help of M‐CP, the OVA peptides were released partly from the lysosomes, accompanied by the incomplete co‐location of OVA and lysosome at 4 h (Pearson's correlation = 0.66, Figure S9h, Supporting Information). The robust FAM fluorescence signal on BMDC cell membranes at 24 and 48 h also supported the phenomena of releasing and further presentation of OVA peptide (Figure 3d).

To further evaluate the efficiency of antigen presentation, 25‐D1.16 antibodies against OVA‐H2kb complexes (OVA peptide‐MHCI complexes) were used to stain BMDCs.^[^ ^2a^ ^]^ In the MCN‐NPs treatment group, the antigen presentation efficiency gradually increased with time (Figure 3e), reaching 8.0‐fold and 2.3‐fold greater than the free OVA and CpG‐OVA treatment group at 48 h (Figure 3f), respectively. However, the MCN‐NPs_(click)_ assembled by irreducible CpG‐OVA conjugate (CpG‐OVA_(click)_) and M‐CP only induced an 8.5% antibodies labeling rate, which was significantly lower than the MCN‐NP group (Figure 3f), and once again proved the importance of restorable sulfonium on CpG‐OVA conjugate.

Furthermore, the maturation of immune cells and secretion of cytokines also proved the effective co‐delivery of antigens and adjuvants using the M‐CP nano‐carrier. After 24 h incubation, MCN‐NPs stimulated potent BMDC maturation by enhancing the expression of DC‐maturing markers, including CD80 and CD86 (Figure 3g–i). In addition, increased cytokine secretion was detected in the culture supernatants of RAW264.7, BMDCs, and peritoneal macrophages with the MCN‐NPs treatment using the ELISA kits (Figure S10, Supporting Information). Particularly, the concentrations of IL‐6, IL‐12, and TNF‐α in MCN‐NPs‐treated BMDCs culture supernatant were 3.6, 6.1, and 176.2‐fold higher than the M‐CP‐CpG group (without antigen, Figure S10d–f, Supporting Information), respectively. These results were consistent with many previous studies, in which the combination of adjuvants and antigens could greatly promote the activation of antigen presentation cells (APCs).^[^ ^8^, ^15^ ^]^ Taken together, all the results above suggested that M‐CP would be an excellent nano‐carrier candidate for the co‐delivery of antigens and nucleic acid‐based adjuvants, and further inducing immune activation.

### 2.4 MCN‐NPs Upregulate the Transcription of Antigen Presentation‐Related Genes and Activate the TLRs Pathways

To monitor the uptake behaviors of nano‐carriers, DC2.4 cells were pre‐treated with different endocytosis inhibitors. We observed that multiple energy‐dependent pathways were simultaneously involved in the internalization of nano‐vaccines to varying degrees (Figure S11a, Supporting Information). Especially, after pretreating with cytochalasin D, chlorpromazine, and genistein, the uptake rate of DC2.4 cells to MCN‐NPs decreased by 79%, 50%, and 49% (Figure S11b, Supporting Information), respectively. These results indicated that nano‐vaccines mainly enter the APCs by micropinocytosis/phagocytosis‐mediated pathways. Meanwhile, clathrin and caveolae‐mediated endocytosis also participated in this internalization process, which was similar to the membrane penetration pathway of previously reported nanoparticles.^[^ ^2^, ^16^ ^]^ To further evaluate the lysosomal escape behavior of nano‐carriers, acridine orange staining experiments were carried out (Figure S11c, Supporting Information).^[^ ^17^ ^]^ Strong red fluorescence was observed in the cytoplasm of RAW264.7 cells in OVA, CpG‐OVA, and CpG‐OVA(Lipo2000) treatment groups (Figure S11d, Supporting Information). In contrast, a much weaker red fluorescence accompanied by a stronger green signal was observed in the cytoplasm of MCN‐NPs‐treated RAW264.7 cells (Figure S11d, Supporting Information). These phenomena were also found in DC2.4 cells (Figure S11e, Supporting Information), indicating that sulfonium‐based nano‐carrier facilitated antigen escape from the endosome/lysosome. Furthermore, the previously mentioned partial co‐location of OVA peptide with lysosome and effective antigen presentation also supported this point.

Next, we analyzed the transcriptional changes of immune pathway‐related genes caused by nano‐vaccines to elucidate its possible mechanism in vitro. Compared with the control, CpG+OVA, CpG‐OVA, and M‐CP treatment groups, 646 differentially expressed genes were detected in the MCN‐NPs treatment group and majorly enriched in immune activation‐related pathways (Figure 3j,k). Further analysis revealed 16 genes were involved in the antigen processing and presentation pathways (Figure S12a, Supporting Information). The qPCR results also supported the sequencing results that the major histocompatibility complex (MHC) class I genes (*H2‐K1*, *H2‐M2*, *H2‐Q7*, and *H2‐T22*) and transporter‐associated proteins of antigen processing genes (*Tap1*, *Tap2*, and *Tapbp*) were significantly upregulated in MCN‐NPs treated cells (Figure S12b,c, Supporting Information), indicating that the nano‐carriers promoted the presentation of antigen peptides.

In addition to efficient antigen presentation on APCs, the activation of downstream immune pathways induced by immune adjuvants was equally important.^[^ ^18^ ^]^ As a ligand for the toll‐like receptor 9 (*TLR9*),^[^ ^19^ ^]^ CpG adjuvant significantly upregulated the transcription of the TLR9 and TLR pathway connector (*MyD88*) genes with the assistance of nano‐carriers (Figure 3l, and Figure S13a, Supporting Information). While, the lower *TLR9* transcription levels in the MCN‐NPs group might be due to the longer time consumption of GSH‐induced nanoparticle disintegration (Figures S5b and S7b, Supporting Information). Furthermore, we also found that the transcriptions of other TLRs (*TLR1*, *2*, *4*, and *6*) were strongly upregulated by MCN‐NPs, especially *TLR2* receptors on the cell membrane (Figure 3l, and Figure S13a, Supporting Information). Thus, we speculated that the toll‐like receptors on the surface of APCs might participate in the uptake of MCN‐NPs and synergistic activation of APCs, as the previous studies have shown that positively charged PEI and some lipopeptides could promote the activation of immunocytes through the toll‐like receptors‐mediated pathway (TLR).^[^ ^2^, ^20^ ^]^ To verify this hypothesis, anti‐TLR antibodies were pre‐incubated with peritoneal macrophages to block the toll‐like receptors on the cell membrane. However, compared to the group without antibody pretreatment (MCN‐NPs), the group pretreated with antibodies did not significantly reduce the uptake of FAM‐labeled MCN‐NPs (Figure S13b, Supporting Information). These results indicated that in the early stages of incubation, the TLR pathway on the cell membrane might not be involved in the internalization of nano‐vaccine. Putting these cellular results together, we speculated that the mechanism of this sulfonium‐based nano‐carrier might involve the following pathways (Figure 3m): MCN‐NPs were internalized in APCs by varying phagocytosis‐mediated pathways. With the help of M‐CP and the disassembly of nano‐carriers induced by GSH, the internal antigens escaped from the endosome/lysosome and were further presented on the surface of APCs. The released CpG adjuvant induced the *TLR9* pathway activation and promoted cytokines secretion and DCs maturation. Meanwhile, MCN‐NPs assist in stimulating a stronger immune response by upregulating the gene transcription of antigen presentation‐related pathways and other TLR pathways, which might further induce a strong and effective T cell response.

### 2.5 MCN‐NPs Effectively Deliver Antigens to dLNs and Elicit the Antigen‐Specific Response

**Figure 4:**
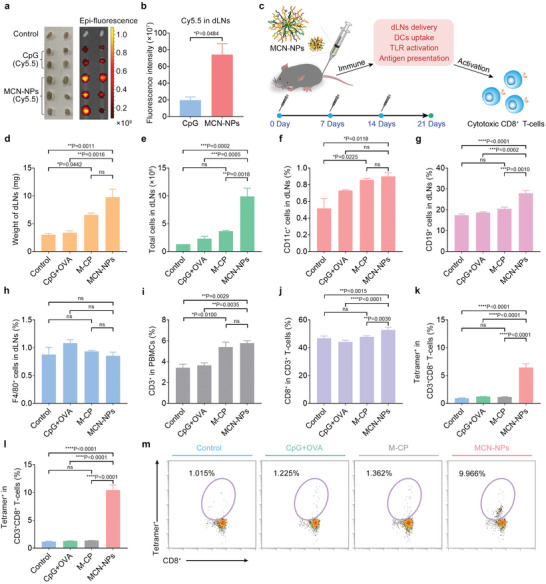
MCN‐NPs promote antigen enrichment in dLNs and elicit specific cytotoxic lymphocyte responses. a) The fluorescence signals and b) intensity statistics of the inguinal draining lymph nodes (dLNs) were quantified with IVIS 24 h after subcutaneous injection with 15 nmol free CpG or MCN‐NPs (Cy5.5 labeled CpG) at the tail base of C57BL/6 mice. c) Schematic illustration showing the potential immune activation mechanism of MCN‐NPs in vivo and the administration plan to evaluate the effectiveness of nano‐vaccines (MCN‐NPs). C57BL/6 mice were immunized with different formulations (15 nmol CpG and 15 nmol OVA antigen) at days 0, 7, and 14. d) The weights of dLNs, e) total cell counts in dLNs, and the percentage of f) DC cells with anti‐CD11c antibody staining, g) B cells with anti‐CD19 antibody staining, and h) macrophages with anti‐F4/80 antibody staining in dLNs at day 21 after three immunizations. i) The percentage of CD3^+^ T cells in PBMCs (peripheral blood mononuclear cells) and j) CD8^+^ T cells in CD3^+^ T cells at day 21. The percentage of SIINFEKL‐specific CD8^+^ T cells in CD8^+^CD3^+^ T cells (Tetramer^+^CD8^+^ T cells) at k) days 15 and l) 21 which corresponded to 1 and 7 days after three immunizations. m) The representative scatter plots of SIINFEKL‐specific CD8^+^ T cells in peripheral blood at day 21. The data were presented as mean ± SEM (*n* = 3) from three independent experiments. Data were analyzed by one‐way ANOVA with Turkey multiple comparisons post‐test (*p < 0.05, **p < 0.01, ***p < 0.001, ****p < 0.0001).

To extensively validate the effectiveness of nano‐carriers for the co‐delivery of antigens and adjuvants, the metabolism of nano‐vaccines was monitored in vivo. Considering the similar assembly characteristics of CpG and CpG‐OVA conjugate with M‐CP (Figure 2f, and Figures S3h and S6d, Supporting Information), a mimic nano‐vaccine assembly from Cy5.5 labeled CpG and M‐CP carriers was subcutaneously injected into the C57BL/6 mice at the tail. After 24 h, the faint Cy5.5 fluorescence was detected in the inguinal dLNs (draining lymph nodes) of free CpG immunized mice (**Figure** **4**a), possibly due to the non‐specific binding at the injection site. In contrast, the MCN‐NPs(Cy5.5) immunized group presented an obvious increase in enrichment of Cy5.5 fluorescence in dLNs (Figure 4a,b), showing the critical effect of M‐CP carrier for antigen and adjuvant co‐delivery. It should be noted that, in addition to liver enrichment of nano‐vaccines like most nanoparticles, the intense Cy5.5 fluorescence in the lung (one of the main immune organs) presented by the MCN‐NPs treatment group was also investigated (Figure S14, Supporting Information). We speculated that the lung enrichment of nano‐vaccines might be propitious to further immune response. Furthermore, the long‐term impact of nano‐vaccines on the immune response in dLNs was also detected. We observed that MCN‐NPs induced the most significant increase in the weights and total cell numbers of dLNs after three immunizations (Figure 4c–e, and Figure S15a,b, Supporting Information), and accompanied by the highest proportion of DC cells (CD11c^+^ cells) and B cells (CD19^+^ cells) (Figure 4f,g). Although no significant proportion change in macrophages (F4/80^+^ cells) was detected (Figure 4h), the data above has sufficiently proved that the MCN‐NPs could remarkably promote the delivery of antigens and adjuvants to dLNs and greatly induce the immune cells activation in vivo. These also provided essential conditions for the subsequent antigen cross‐presentation and antigen‐specific T cell response.

To verify the specificity of T cell response, OVA peptide, utilized as a model neoantigen, was packed into a nano‐carrier for mice immunization. After three immunizations of OVA‐contained nano‐vaccines in a 7‐day interval, the peripheral blood was acquired and further analyzed for the percentage of CD8^+^ T cells and H2kb OVA tetramer^+^ CD8^+^ T cells by antibody staining. The results illustrated the mixture of OVA peptide and CpG adjuvant (CpG+OVA) induced 1.2% and 1.3% OVA‐specific CD8^+^ T cells 1 and 7 days after the third immunization (Figure 4k,l, and Figure S15e,f, Supporting Information), respectively. In contrast, MCN‐NPs not only remarkably induced a higher proportion of CD3^+^ and CD8^+^ T cells in PBMCs (peripheral blood mononuclear cells) at day 21 (Figure 4i,j) but also elicited 10.5% OVA‐specific CD8^+^ T cells, reaching 8.2‐fold higher than the CpG+OVA treatment group (Figure 4l,m). These results demonstrated that with the delivery of M‐CP, OVA_257‐274_ peptide (an MHC I‐restricted T cell epitope of OVA protein^[^ ^21^ ^]^) greatly promoted the antigen‐specific CD8^+^ T cells activation. Under the robust CD8^+^ T cells response, the proportion of CD4^+^ T cells slightly decreased in the MCN‐NPs immunized group, this might be due to the expansion inhibition of Treg cells (Figure S15c,d, Supporting Information). Furthermore, the safety of peptide carriers was evaluated by detecting the antibodies against the M‐CP or M‐LP in serum at day 21. Throughout the studies, no autoimmunity signal was detected in M‐CP or MCN‐NPs treatment groups (Figure S16a,b, Supporting Information). The results above indicated that M‐CP could be used as a bio‐safe and effective nano‐carrier for the co‐delivery of neoantigens and adjuvants to stimulate robust and sustained antigen‐specific responses.

### 2.6 Activated CTLs Greatly Suppress Tumor Metastasis and Prolong Survival

**Figure 5:**
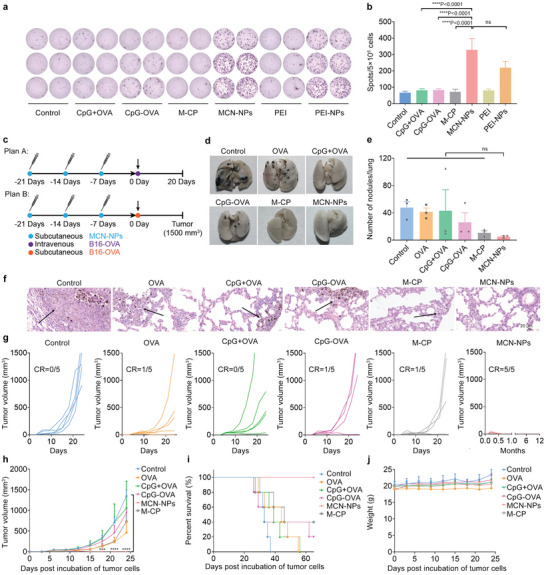
The strong CTL response induced by MCN‐NPs inhibits tumor metastasis and growth. a) ELISPOT analysis and b) spot statistics of IFN‐γ spot‐forming cells among 5×10^5^ splenocytes after restimulation with OVA peptide at day 7 post‐immunization (*n* = 3). 25 kDa branched‐PEI and PEI‐NPs (nanoparticles assembled from 80 µg PEI and 15 nmol CpG‐OVA conjugate) were utilized as the positive group as described in the previous report. c) Administration plans for tumor immune prevention. C57BL/6 mice were subcutaneously injected with different formulations (15 nmol CpG and 15 nmol OVA antigen) at days 21, 14, and 7 at the tail base. For the pulmonary metastasis model (plan A, *n* = 3), pre‐immunized mice were intravenously challenged with 5 × 10^5^ B16‐OVA tumor cells at day 0 and sacrificed at day 20. For the tumor growth inhibition model (plan B, *n* = 5), pre‐immunized mice were subcutaneously seeded with 1 × 10^6^ B16‐OVA cells at the right flank at day 0, and tumor growth was continuously monitored. d) The pictures, e) numbers of metastatic nodules, and f) H&E‐stained sections of lungs at day 20 (plan A). Scale bar = 20 µm. g) The individual and h) average tumor growth curves of the B16‐OVA tumor (plan B). CR, tumor complete response rate. i) The survival rate of different immunized groups of mice with B16‐OVA tumors (plan B). j) Weight growth curves of the tumor‐bearing mice (plan B). The data were presented as mean ± SEM (*n* ≥ 3). Data were analyzed by one‐way ANOVA with Turkey multiple comparisons post‐test or two‐way ANOVA with Bonferroni post‐test (*p < 0.05, **p < 0.01, ***p < 0.001, ****p < 0.0001).

The secretion of IFN‐γ is a direct indicator for evaluating the tumor‐killing ability of T cells.^[^ ^2a^ ^]^ To evaluate the specificity and effectiveness of the anti‐tumor capability of nano‐vaccines, splenic lymphocytes from the immunized C57BL/6 mice at day 21 were harvested and re‐stimulated by OVA peptides (Figure S15a, Supporting Information). As the positive group, mice were vaccinated with an equal amount of CpG‐OVA conjugates wrapped up into the branched PEI carrier (one of the most potential carriers with adjuvant properties in peptide‐based cancer vaccines^[^ ^8^, ^20^ ^]^). We discovered that the re‐stimulation of splenocytes from the MCN‐NPs treated mice induced the maximum IFN‐γ^+^ immune spots (**Figure** **5**a), showing 1.5 and 4.1 times higher than the PEI‐NPs and CpG+OVA immunized groups (Figure 5b), respectively. Moreover, a slight proliferation of splenic lymphocytes was detected in the MCN‐NPs immunized group (Figure S16c, Supporting Information). Combined with the robust OVA‐specific CD8^+^ T cells activation in PBMCs (Figure 4k,l), these results again demonstrated that the MCN‐NPs nano‐vaccines successfully elicited the antigen‐specific and cytotoxic T cell (CTL) immune responses in vivo.

Next, the anti‐tumor efficiency of MCN‐NPs was detected in two types of tumor prevention models. In the lung metastasis model, the pre‐immunized C57BL/6 mice were intravenously injected with 5 × 10^5^ B16‐OVA tumor cells which stably expressed OVA proteins (Figure 5c, Plan A), and were sacrificed 20 days post‐vaccination. We observed that nano‐vaccines intensely induced an anti‐tumor metastasis effect, which was manifested by the significantly lowered melanoma nodule numbers and nearly undetectable melanoma‐infected pneumonocytes in lungs up to 20 days after tumor seeding (Figure 5d–f). Whereas, the control, OVA peptide, the mixture of CpG and OVA, and free CpG‐OVA conjugate immunization group only partially inhibited the lung metastasis of melanoma (Figure 5e). A similar tumor prevention effect was also observed in the subcutaneous tumor model of B16‐OVA. 7 days after the third immunization, mice were subcutaneously injected with 1 × 10^6^ B16‐OVA cells/mouse (Figure 5c, plan B). Notably, mice that were pre‐vaccinated with MCN‐NPs completely rejected the growth of melanoma, and no tumor recurrence was observed for more than 11 months (Figure 5g,h). Meanwhile, compared to the free antigen/adjuvant vaccines, the survival rate in the MCN‐NPs immunized group dramatically extended to 65 days (Figure 5i). These findings demonstrated that the nano‐vaccines based on sulfonium peptide might provide a new option for the long‐term prevention of postoperative tumor recurrence. From the two tumor prevention models, MCN‐NPs showed more significant tumor prevention effects than the other groups. This might be attributed to the stronger dLNs enrichment induced by MCN‐NPs (Figure 4a), which further elicit higher proliferation rates of OVA‐specific CD8^+^ T cells in peripheral blood (Figure 4k–m) and more activation of CTLs in the spleen (Figure 5a,b). Importantly, throughout the tumor challenge tests, we did not observe any obvious signs of organ damage, inflammatory lesions, or significant weight loss according to the H&E‐stained sections (Figure S16d, Supporting Information) and statistical results of immunized mice weight (Figure 5j). Especially, compared with the low toxicity of high‐dose PEI,^[^ ^20^, ^22^ ^]^ our nano‐vaccine demonstrated its potential application as a safe and effective neoantigen‐based vaccine in clinical trials. It should be noted that free M‐CP showed certain tumor prevention effects in both tumor models. We speculated that this might be due to the non‐immune responses induced by M‐CP.

### 2.7 MCN‐NPs Precisely Inhibit Tumor Growth and Adjust Tumor Infiltration Microenvironment

**Figure 6:**
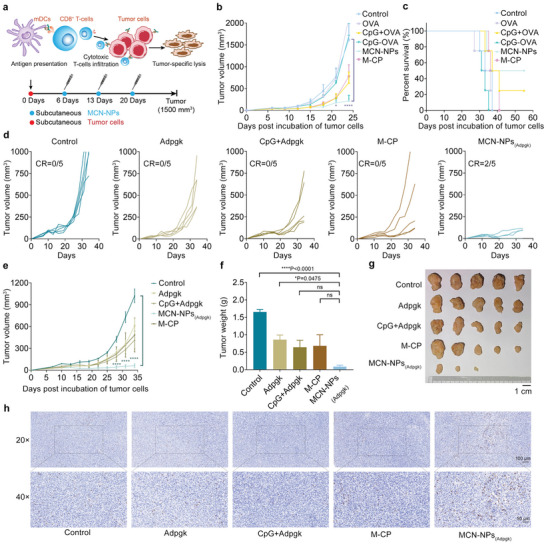
MCN‐NPs for personalized tumor immunotherapy. a) Schematic diagram of tumor‐specific elimination induced by MCN‐NPs vaccines and the administration plan of nano‐vaccines (MCN‐NPs) for tumor immunotherapy. C57BL/6 mice were subcutaneously injected with 1 × 10^6^ B16‐OVA cells at day 0, and vaccinated with the indicated formulations (15 nmol CpG and 15 nmol OVA antigen) at days 6, 13, and 20 at the tail base. Shown are b) the average tumor growth curves of the B16‐OVA tumor and c) the survival curves of tumor‐bearing mice (*n* = 4). d) The individual and e) average tumor growth curves of the MC‐38 tumor (*n* = 5). After the subcutaneous challenge of 1 × 10^6^ MC‐38 tumor cells at day 0, mice were vaccinated with the indicated formulations (15 nmol CpG and 15 nmol Adpgk neoantigen) three times with 7‐day intervals (days 7, 14, and 21), and sacrificed until the tumor volume reached to 1000 mm^3^. CR, tumor complete response rate. f) the weight and g) pictures of the MC‐38 tumor at day 34. Scale bar = 1 cm. h) Representative immunohistochemistry (IHC) images of MC‐38 tumor showing CD8^+^ T cells infiltration in the tumor tissues. Blue represented cell nuclei. Brown represented CD8^+^ T cells which were labeled by anti‐CD8 antibodies. The data were presented as mean ± SEM (*n* ≥ 3). Data were analyzed by one‐way ANOVA with Turkey multiple comparisons post‐test or two‐way ANOVA with Bonferroni post‐test (*p < 0.05, **p < 0.01, ***p < 0.001, ****p < 0.0001).

Previous studies have pointed out that one of the greatest advantages of neoantigen‐based vaccines is to respectfully conquer solid tumors.^[^ ^1c^ ^]^ To further evaluate the efficacy of MCN‐NPs as a therapeutic vaccine, the tumor‐bearing mice models were carried out. Before nano‐vaccine treatment, mice were subcutaneously inoculated with 1 × 10^6^ B16‐OVA cells on the flank at day 0 (**Figure** **6**a). Six days later, the indicated vaccines were subcutaneously injected into the tumor‐bearing mice at 7‐day intervals (Figure 6a). The statistical data illustrated that free OVA peptide and CpG‐OVA conjugate did not provide any benefit for tumor growth suppression or mice survival prolongation compared with the control group (Figure 6b,c). The mixture of CpG and OVA led to moderated melanoma suppression (Figure 6b, and Figure S17a–f, Supporting Information). In contrast, MCN‐NPs nano‐vaccines induced the most significant tumor growth inhibition and complete tumor regression in 25% of tumor‐bearing mice (Figure S17a–f, Supporting Information). Further analysis revealed that no tumor recurrence was observed in these mice for more than 55 days (Figure 6c), indicating the potential application of this nano‐vaccine for tumor immunotherapy.

To further estimate the universality of this sulfonium‐based stapling peptide nano‐carrier platform in the development of therapeutic vaccines against different neoantigens, we replaced the model OVA antigen in MCN‐NPs vaccine with Adpgk mutant peptide (neoantigen screened from MC‐38 tumor^[^ ^2a^ ^]^). To imitate the true tumor situation, mice were subcutaneously inoculated with 1 × 10^6^ MC‐38 tumor cells (cell line of murine carcinoma of the colon without exogenous OVA protein expression) on the flank at day 0. The MCN‐NPs_(Adpgk)_ nano‐vaccines were synthesized by mixing M‐CP carrier with CpG‐Adpgk conjugate, and immunized MC‐38 tumor‐bearing mice at days 7, 14, and 21 (Figure S18a, Supporting Information). A similar anti‐tumor effect was detected in MC‐38 tumor‐bearing mice treated with MCN‐NPs_(Adpgk)_, showing a substantial decrease in tumor growth and minimized average tumor weight at day 34 (Figure 6d–g). Notably, different from Adpgk, CpG+Adpgk, and M‐CP carrier peptide, MCN‐NPs_(Adpgk)_ induced complete tumor regression in 40% of tumor‐bearing mice (Figure 6d,g). Although there was no significant statistical difference in tumor weight between the CpG+Adpgk/M‐CP and MCN‐NPs groups, this was mainly due to individual differences within the group caused by complex immune responses. In addition, complete tumor regression in 40% of tumor‐bearing mice and the most significant tumor growth inhibition were found only in the MCN‐NPs treatment group, indicating the effectiveness of the MCN‐NPs vaccine in tumor immunotherapy.

Furthermore, we found that free peptide and soluble CpG+Adpgk only induced 1.74% and 0.89% of CD8^+^ T cells infiltration in MC‐38 tumor tissue, which was similar to the control group (1.86%) (Figure 6h, and Figure S18c, Supporting Information). In contrast, vaccinations with MCN‐NPs_(Adpgk)_ elicited more significant immune cells infiltration in tumor tissue (4.94%), reaching a 5.6‐fold increase in CD8^+^ T cells proportion compared with the CpG+Adpgk treatment group (Figure 6h, and Figure S18c, Supporting Information). Predictably, the more immune cells infiltrate the tumor microenvironment, the deeper the induction of tumor cell apoptosis. The apoptotic tumor cell fragments will further be taken up by APC cells, activating more tumor‐specific T cell activation, and eliciting a larger area of tumor killing. At this moment, MCN‐NPs act as an inducer of closed‐loop response in tumor immunotherapy. Taken together, our results highlighted the sulfonium peptide‐based nano‐vaccines’ potential in regulating the tumor microenvironment and solid tumor treatment. In addition, throughout the experiment, nano‐vaccines did not show any observable toxicity toward major organs or effects on the mouse body weight (Figures S17g,h and S18b, Supporting Information), which was consistent with the tumor prevention studies above (Figure 5j, and Figure S16d, Supporting Information).

In addition, compared with the PBS treatment group, free M‐CP peptide vaccination elicited moderated tumor metastasis and growth inhibition to a certain extent (Figures 5e,g–i and 6b,d,e). This phenomenon was consistent among the four tumor models. Thus, we speculated that M‐CP might induce non‐specific immune responses in vivo as a novel adjuvant. As shown in Figure 2e and Figure S1, Supporting Information, M‐CP is positively charged. Two sulfonium centers on the side chain further increased the positivity of M‐CP. Previous studies have pointed out that the positively charged PEI and cationic liposome carriers could act as vaccine adjuvants by activating the nonspecific immune responses.^[^ ^2^, ^23^ ^]^ Therefore, M‐CP might exert its adjuvant properties through the positive charge and induce non‐specific anti‐tumor therapeutic effects, which are currently being validated. In a word, our results have sufficiently proved that the efficient delivery of neoantigen peptides (even with only one peptide) by a suitable carrier could elicit a strong immune response and significant tumor regression. Even if MCN‐NPs did not induce great tumor regression in tumor‐bearing mice, we could reasonably speculate that the nano‐vaccine proposed in this study will provide a simple, safe, and effective neoantigen and adjuvant co‐delivery platform for personalized tumor immunotherapy. In addition, the combination of MCN‐NPs with various antigens (like shared‐neoantigens and tumor‐associated antigens) or checkpoint inhibitors (like anti‐PD‐1 and anti‐CTRL‐4 antibodies^[^ ^24^ ^]^) could be considered as the next step to improve the therapeutic effect for personalized tumor immunotherapy.

## 3 Conclusion

In summary, we have developed a novel nano‐vaccine containing neoantigen peptide based on the nucleic acid‐induced self‐assembled stapling peptide platform. This nano‐vaccine not only facilitated the co‐delivery of neoantigen peptides and adjuvants but also activated the antigen processing and TLRs pathways, thus resulting in broad and effective anti‐tumor immune response. We have demonstrated that, by cooperating with efficient carriers, a single neoantigen peptide could sufficiently trigger strong cytotoxic T lymphocyte infiltration in tumor tissues, achieving tumor‐specific elimination. Furthermore, the sulfonium‐based peptide carriers with only 9 amino acids have demonstrated their advantages in promoting membrane penetration and lysosomal escape for downstream antigen presentation of neoantigen peptides. Considering the rapid and facile preparation process, low cost, and excellent biocompatibility of peptide materials, our stapling peptide‐based nano‐vaccine could provide an attractive candidate for personalized tumor immunotherapy in clinical trials. Moreover, by simply replacing the internal antigens, this nano‐vaccine can quickly expand its applications for different tumor treatments, stating great potential in preparing universal tumor vaccines based on the “shared” neoantigen. Besides the short‐stranded nucleic acid or conjugate delivery demonstrated in this study, the stimuli‐responsive nanoplatform might also open a new avenue for mRNA vaccines, for which efficient delivery and lysosome escape are essential to elicit a broad immune response.

## 4 Experimental Section

Details on materials and methods used are provided in the Supporting Information, covering the following sections: Materials, Animals and Cell lines, Construction of Computational Model, Simulation Setup, Simulation Data Analysis, Synthesis of the Stapling Peptides, Synthesis of Neoantigen with Propargyl Sulfonium Center, Synthesis and Characterization of CpG‐neoantigen Conjugates, Characterization and Optimization of MCN‐NPs, Reduction of Conjugate and Disassemble Nanoparticles by GSH, Cytotoxicity Assessment in vitro, Cell Uptake and Intracellular Localization of MCN‐NPs, Presentation of Antigen, BMDCs Activation and Immune Factor Secretions Assessment, Analysis of Inhibition of Endocytosis and TLRs Pathway, Transcriptome Sequencing Analysis and Verification, dLNs Analysis, In Vivo Immunization and Phenotypic Assessment of T cells, Immunogenicity Assessment of Peptide Carriers, Immune Oncology Prevention, Tumor Immunotherapy, and Statistical Analysis. Supporting Figures and Tables are also provided, as referenced in the text.

## Conflict of Interest

The authors declare no conflict of interest.

## Supporting information

Supporting Information

## Data Availability

The data that support the findings of this study are available from the corresponding author upon reasonable request.
