# Supplementary material for: Sulfonium‐Stapled Peptides‐Based Neoantigen Delivery System for Personalized Tumor Immunotherapy and Prevention
Source: Adv Sci (Weinh). 2024 Apr 11;11(24):2307754. doi: 10.1002/advs.202307754 (PMC11200081; doi:10.1002/advs.202307754)
Supplement: Supplementary file 1 — Supporting Information [file ADVS-11-2307754-s001.pdf]

ADVANCED SCIENCE
Open Access

Supporting Information

for *Adv. Sci.*, DOI 10.1002/advs.202307754

Sulfonium-Stapled Peptides-Based Neoantigen Delivery System for Personalized Tumor Immunotherapy and Prevention

Yaping Zhang, Leying Jiang, Siyong Huang, Chenshan Lian, Huiting Liang, Yun Xing, Jianbo Liu, Xiaojing Tian, Zhihong Liu, Rui Wang, Yuhao An, Fei Lu, Youdong Pan, Wei Han, Zigang Li and Feng Yin**

Supporting Information

Sulfonium-stapled peptides-based neoantigen delivery system for personalized tumor immunotherapy and prevention

Yaping Zhang, Leying Jiang, Siyong Huang, Chenshan Lian, Huiting Liang, Yun Xing, Jianbo Liu, Xiaojing Tian, Zhihong Liu, Rui Wang, Yuhao An, Fei Lu, Youdong Pan, Wei Han, Zigang Li, and Feng Yin**

Y. P. Zhang, C. S. Lian, H. T. Liang, Y. Xing, J. B. Liu, Z. H. Liu, R. Wang, Y. H. An, Z. G. Li, F. Yin
Pingshan Translational Medicine Center, Shenzhen Bay Laboratory, Shenzhen 518055, P.R. China
E-mail: lizg@pkusz.edu.cn; yinfeng@pkusz.edu.cn

L. Y. Jiang, S. Y. Huang, X. J. Tian, F. Lu, W. Han, Z. G. Li
State Key Laboratory of Chemical Oncogenomics, School of Chemical Biology and Biotechnology, Peking University Shenzhen Graduate School, Shenzhen 518055, P.R. China

Y. D. Pan
NeoCura Bio-Medical Technology Co., Ltd., Shenzhen 518055, P.R. China

Experimental Section/Methods

Materials: Antigen peptides used in this study include ovalbumin antigen peptides $OVA_{257-264}$ (SIINFEKL, SIINFEKL-RRW(L-Pra) for CpG-OVA conjugate preparation based on click chemistry, WMβAla-SIINFEKL and MβAla-$SIINFEK_{(FAM)}$L for the preparation of CpG-OVA conjugate with sulfonium) and Adpgk mutant peptide from MC-38 tumor (ELASMTNMELMSS). All antigen peptides were obtained from QYAOBIO (Shanghai, China). CpG_{1826} single-stranded DNA was from Invitrogen (Shanghai, China). CCK8 kits, Gel Red, and Hoechst33342 were from Solarbio Life Science (Beijing, China). DAPI, Lyso-Tracker Red DND-99, Lipofectamine 2000, acridine orange solution, and flow cytometry staining buffer were purchased from Thermo Scientific (Pittsburgh, PA, USA). IL-4, GM-CSF, cytochalasin D, methyl-β-cyclodextrin, genistein, amiloride, 2-deoxyglucose, and chlorpromazine were purchased from MedChemExpress (New Jersey, USA). IL-12, TNF-α, and IL-6 kits were purchased from Elabscience Biotechnology Co. Ltd. (Wuhan, China). One-step prime script RT-PCR kits were purchased from Takara Biomedical Technology (Beijing, China). The Mouse IFN-γ ELISPOT kit was purchased from MABTECH (Sweden). Evo M-MLV RT Master Mix kit was purchased from Accurate Biotechnology (Hunan) Co. Ltd. (ChangSha, China). 7-AAD kit was purchased from Biolegend (San Diego, USA). Mouse TLR1 (Cat. No. NB100-56563), TLR2 (Cat. No. NB100-56722), TLR5 (Cat. No. NBP2-24787), and TLR6 (Cat. No. NBP1-54336) antibodies were purchased from Novus Biologicals (USA). Mouse TLR4 antibody (MTS510, Cat. No. sc-13591) was from Santa Cruz Biotechnology (USA). PE-conjugated anti-mouse SIINFEKL/H-2Kb monoclonal antibody 25-D1.16 (Clone 25-D1.16), CD16/CD32 (Clone 93), APC-conjugated CD11c (Clone N418), PE-conjugated CD19 (Clone 6OMP31), APC-conjugated F4/80 (Clone BM8), FITC-conjugated CD80 (Clone 16-10A1), PE-conjugated CD86 (Clone GL1), eFluor450-conjugated CD3e (Clone 145-2C11), FITC-conjugated CD8a (Clone 53-6.7), APC-conjugated CD4 (Clone RM4-5) anti-mouse antibodies were purchased from Thermo Scientific (Pittsburgh, PA, USA). APC-conjugated H2kb OVA tetramer (Cat. No. TS-5001-1C) was purchased from MBL (Beijing, China). Lysing buffer 10× was purchased from BD (USA). All other chemicals were purchased from Sigma-Aldrich (St. Louis, MO, USA).

Animals and Cell Lines: 293T, HeLa, RAW264.7, and MC-38 cell lines were purchased from ATCC. DC2.4 and B16-OVA cells were gifts from Prof. Xingjie Liang (the National Center for Nanoscience and Technology, Beijing, China). BMDCs (immature antigen-presenting cells)

were isolated from the bone marrow of C57BL/6 mice as described in the previous reports.[1] Peritoneal macrophages were separated from the abdominal cavity of C57BL/6 mice after three days of 6% starch broth solution (1 mL per day, containing 6% soluble starch, 0.3% beef extract, 1% peptone, and 0.5% NaCl.) stimulation via abdominal injections. RAW264.7, DC2.4, BMDCs, peritoneal macrophages, and B16-OVA cells were cultured in RPMI1640 complete medium, containing 10% fetal bovine serum at 37°C in 5% CO_2. The culture medium used for HeLa, 293T, and MC-38 cells was DMEM, added 10% FBS, streptomycin (100 μg mL^{-1}), and penicillin (100 U mL^{-1}). All cell lines were identified by the short tandem repeat method and no mycoplasma or rodent pathogen infection was detected. In this study, female C57BL/6 mice (6-8 weeks) were provided by Guangdong Vital River Laboratory Animal Technology Co., Ltd. (Guangdong, China). All tests related to animals were conformed to the Care and Use of Laboratory Animals guidelines issued by the National Institutes of Health and approved by the Animal Care and Use Committee of Peking University (Shenzhen, SYXK (Yue) 2017-0172).

Construction of Computational Model: Here, we used an all-atom model to simulate the self-assembly of M-CP/5A or M-LP-S^+/5A. The Fmoc group was parameterized using the parameters developed by T. Tuttle,[2] which were modelled to reproduce binding energies of Fmoc-Fmoc and Fmoc-Water dimer. These parameters were provided in NAMD format and converted to GROMACS format. The short peptide composed of natural amino acids and the nucleic acid portion were described by the CHARMM36m force field.[3] The bridging of two methionine residues was parameterized using parameters from CHARMM36m, where the benzene ring was modelled using parameters from the PHE side chain and the positively charged sulfonium atom was modelled using the parameters from S-adenosylmethionine in CGenFF.[4] Based on the wet-lab test (N/P = 4) and experience, seventy-two M-CP or M-LP-S^+ molecules and twelve 5A molecules were randomly put in a 10×10×10 nm cubic box and solvated with three-site transferable intermolecular potential (TIP3P) water molecules. Cl^- ions were added to neutralize the charge of the system.

Simulation Setup: All-atom molecular dynamics simulations were performed using the GROMACS package (version 2022).[5] The Particle-Mesh Ewald (PME) algorithm was used for calculating the electrostatic interactions with a nonbonded cutoff of 10 Å. The time step was set to 2 fs with a linear constraint solver (LINCS). The simulations were performed at 310 K and 1.0 atm in the isothermal-isobaric (NPT) ensemble, using the v-rescale method and the

Parrinello-Rahman method to keep a constant temperature and pressure, respectively. For each system, 1000 ns of the production run was performed after equilibration.

Simulation Data Analysis: The trajectories were extracted and cluster analysis was carried out. The dynamic changes in cluster size, composition, structure distribution, and the interaction of each component in the aggregate were analyzed to explore the co-assembly structure from the whole to the part. Two molecules are considered to have contacted and belong to a cluster if there are three or more pairs of non-hydrogen atoms within 4.5 Å of each other. In the whole 1000 ns simulation, the number and the size of the clusters (the total number of molecules in a cluster) were counted every 10 frames, and the average number of clusters in 0-5 ns, 5-500 ns, and 500-1000 ns were calculated. The number of the average contacts in large clusters with size over 60 was also counted every 10 frames in the whole trajectory, that is the average number of one component contacting with the other component. These can be considered as an evaluation indicator of interaction strength. And higher frequencies corresponded to stronger interaction. The atomic contact map was calculated from the frequency of contact between atoms with a distance within 4.5 Å in the last 50 ns simulations. The number of atomic contacts was counted and averaged by the number of M-CP or M-LP-S$^+$ molecules. The contact frequencies of atoms belonging to the same amino acid or nucleotide were summed up.

Synthesis of the Stapling Peptides: The linear M-LP and C-LP peptides were synthesized by the standard Fmoc-based solid-phase peptide synthesis strategy.[6] For the preparation of the stapling peptide with two sulfonium centers (M-CP, M-CP-S'), the M-LP peptides were freeze-dried after HPLC purification and were then dissolved in 10% formic acid buffer (500 μL, acetonitrile: water: formic acid (V:V: V) = 5:4:1), followed by addition of 5 eq of α, α'-dibromo-o-xylene or (E)-1,4-Dibromobut-2-ene. Then the mixtures were placed at room temperature overnight. Unlike M-CP, C-LP reacted with α, α'-dibromo-o-xylene in an alkaline condition (acetonitrile: water: DIPEA (V: V: V) = 5:4:1) for 6 h to obtain C-CP without sulfonium centers. For the preparation of M-LP-S+, the M-LP lyophilized powder was dissolved in 10% formic acid buffer (500 μL, acetonitrile: methyl iodide: formic acid (V: V: V) = 7:3:1) and stayed overnight at room temperature before drying by nitrogen gas. After purification again by HPLC, the peptide carrier candidates were authenticated by LC-MS.

Synthesis of Neoantigen with Propargyl Sulfonium Center: To construct reducible CpG-neoantigen conjugates, the propargyl was introduced to the methionine position of the

neoantigen (ovalbumin antigen peptide WMβAla-SIINFEKL, MβAla-SIINFEK$_{(FAM)}$L, Adpgk mutant peptide ELASMTNMELMSS), which were described in the previous studies.[1a, 7] In detail, antigen peptide was dissolved in 10% formic acid buffer (500 μL, acetonitrile: water: formic acid (V: V: V) = 5:4:1), then 10 eq of 3-bromopropyne was added. The mixture was stirred overnight at room temperature. The products were purified and identified by LC-MS.

Synthesis and Characterization of CpG-Neoantigen Conjugates: The reducible CpG-neoantigen conjugates were prepared with sulfonium-driven DNA and peptide coupling strategy as we previously reported.[1a] CpG (100 μM) with sulfhydryl modification at 5'-end was incubated with 5 eq of propargyl-sulfonium-containing antigen peptide in $(NH_4)_2CO_3$ buffer (10 mM, pH9.6) at 4°C for 4 h. The mixture was analyzed by 10% native PAGE (polyacrylamide gel), purified by HPLC, and further identified by MALDI TOF.

Characterization and Optimization of MCN-NPs: The negatively charged CpG/CpG-neoantigen can induce rapid self-assembly of the positively charged M-CP into regular nanospheres. To evaluate the entrapment rate of the peptide carriers, different concentrations of M-CP (dissolved in ddH_2O) were incubated with CpG (2 μM) or CpG-neoantigen conjugate (dissolved in ddH_2O) at room temperature followed by quick blowing and well mixing. N/P ratio represents the molar ratio of the overall positive charges of M-CP (containing four positive charges in an M-CP molecule which can be originated from three arginines, one histidine, two positive sulfonium centers, and two glutamates) to the negative charges of phosphate from the nucleic acid. The position where the nucleic acid band disappeared in 3% agarose gel was used to determine the entrapment efficiency of M-CP. The zeta potential and particle size were measured by DLS (dynamic light scattering, Malvern Zetasizer, Britain). In addition, the particle size and zeta were also used to evaluate the stability of nanoparticles in 10% serum at 4°C for 24 h. After 24 h incubation with simulated gastric juice (0.3 mg pepsin in PBS, pH2.0), simulated intestinal fluid (0.5 μg trypsin in PBS, pH6.0), and DNase buffer (0.1 U DNase I) at 37°C, the stability of MCN-NPs was evaluated by 3% agarose gel. The micromorphology of nanoparticles was observed by SEM (scanning electron microscope, SUPRA 55 SAPPHIRE, Carl Zeiss, Germany) after drying the sample on a silicon slice. The half of melting temperature value (T_m) and onset aggregation temperature (T_{agg}) were evaluated by high throughput multi-parameter protein stability analyzer (Uncle, Unchained Labs, USA). Herein, peptide intrinsic fluorescence for T_m calculation and static light scattering signal for T_{agg} calculation were collected under the heating program of 25-95°C to estimate the stability of nanoparticles.

Reduction of Conjugate and Disassemble Nanoparticles by GSH: For the reversibility verification of the carrier, different concentrations of GSH (1, 2, 4, 6, 8, 10 mM) were incubated with MCN-NPs ([CpG-OVA]: [M-CP] = 1:300, [CpG-OVA] = 2 μM) in PBS buffer (pH7.4) at 37°C for 24 h. Then the reduction product was subjected to 3% agarose gel. The disassembly of MCN-NPs was calculated based on the appearance and increment of the CpG-OVA band on the gel. For the conjugate reduction, pure CpG-OVA (40 μM) was incubated with GSH (4 mM) at 37°C for 0, 4, 8, 12, 24, 36, 48, and 72 h. The mixture was then analyzed with 10% native PAGE and MALDI TOF to identify the release of CpG from OVA.

Cytotoxicity Assessment in vitro: To assess the safety and biocompatibility of carriers and nano-vaccines, CCK8 and hemolysis tests were carried out. Take RAW264.7 for example, 7000 RAW264.7 cells per well were pre-plated in 96-well plates. After 24 h, the indicated formulations were added and incubated with cells for another 24 h. Then the CCK8 solution (10 μL) was added into each well. After 4-6 h incubation at 37°C, the absorbance at 450 nm was recorded by a microplate reader (BioTek H1, USA). The toxicity of nano-vaccines to DC2.4, 293T, and HeLa cells was tested by the same strategy as described above. In the hemolysis test, PBS, 0.1% Triton X-100, M-CP (300 μM), and MCN-NP (300 μM, [CpG-OVA]: [M-CP] = 1:300) were respectively incubated with 1×10^7 fresh blood cells from mice at 37°C for 1 hour, then the breaking of erythrocyte membrane was evaluated at 570 nm (based on the absorbance of heme).

Cell Uptake and Intracellular Localization of MCN-NPs: The cell uptake of MCN-NPs by immunocytes (DC2.4, RAW264.7, and BMDCs) was evaluated by flow cytometry. Briefly, 5×10^4 cells were plated in 24-well plates and incubated with free OVA peptide, CpG-OVA conjugate, CpG-OVA conjugate mixed with Lipofectamine 2000 (1.5 μL), and MCN-NPs (calculated based on OVA peptide, [OVA] = 1 μM, [CpG-OVA] :[M-CP] = 1:300) at 37°C for 4 h, respectively. Here, the OVA peptide was labeled with FAM dye. After washing 3 times with PBS and quenching extracellular fluorescence with trypan blue, the cells were analyzed by flow cytometer (Attune NxT, Thermo Fisher, USA). In addition, the change of intracellular fluorescence in DC2.4 and BMDCs over time was also monitored by flow cytometry. To visualize the intracellular localization of MCN-NPs in immunocytes, 5×10^4 RAW264.7 cells were plated in 24-well plates and treated with different vaccine formulations at 37°C for 4 h. Cells were fixed with 4% formaldehyde and imaged by a confocal laser scanning microscopy (Nikon A1R, Japan) to determine the localization of antigen peptide. To further evaluate the

lysosomal escape of MCN-NPs, 2.5×10^4 antigen-presenting cells (DC2.4 and BMDCs) seeded in 8-well plates were treated with the indicated formulations at 37°C for 2, 4, 6, 12, 24, and 48 h. Then cells were incubated with Hoechst 33342 (nuclei dye, 3 μg mL^{-1}) and Lyso-Tracker Red DND-99 (lysosomal dye, 200 nM) for 30 min, followed by imaging with confocal laser scanning microscopy.

Presentation of Antigen: In a 24-well plate, 2×10^5 BMDCs were pre-plated and treated with OVA peptide (5 μM), CpG-OVA conjugate (5 μM), MCN-NPs (5 μM, calculated based on OVA peptide), or MCN-NPs$_{(click)}$ (5 μM, the CpG-OVA conjugate in MCN-NPs$_{(click)}$ was prepared based on the classic alkynyl-azide addition[1b]), respectively, for 48 h. To quantify the presentation of antigens on the cell surface, BMDCs were harvested, resuspended in flow cytometry staining buffer (100 μL), and incubated with CD16/CD32 (1 μL) at room temperature for 10 min. The cells were then stained with PE-labeled 25-D1.16 H2kb antibody for 20 min, and analyzed by the flow cytometer. To visualize the presentation, the treated BMDCs were stained with CD16/CD32 and PE-labeled 25-D1.16 H2kb antibodies. After fixating and sealing, BMDCs were imaged by confocal laser scanning microscopy.

BMDCs Activation and Immune Factor Secretions Assessment: 5×10^5 BMDCs were seeded in 24-well plates and stimulated by the physical mixture of OVA and CpG ([CpG] = 1 μM and [OVA] = 1 μM), M-CP (300 μM), and MCN-NPs (1 μM, [CpG-OVA]: [M-CP] = 1:300, [CpG-OVA] = 1 μM) for 24 h, respectively. BMDCs were harvested and incubated with CD16/CD32 (1 μL) at room temperature for 10 min, then the antibody cocktail containing FITC-conjugated CD80, APC-conjugated CD11c, and PE-conjugated CD86 was added. After 20 min incubation, the cells were washed with PBS and analyzed by flow cytometry. For the evaluation of cytokine secretion, RAW264.7, BMDCs, or peritoneal macrophages were incubated with the indicated formulations at 37°C for 24 h, respectively. The cell culture supernatant was collected and detected by TNF-α, IL-6, and IL-12 ELISA kits according to manufacturing instructions.

Analysis of Inhibition of Endocytosis and TLR Pathway: To delve into the cellular internalization mechanism of MCN-NPs, various inhibitors were used to pre-treat DC2.4 cells to inhibit endocytosis.[8] Briefly, DC2.4 cells were incubated with the mixture of 2-deoxyglucose (10 mM) and 0.1% NaN_3 (inhibitor of energy-dependent pathways), cytochalasin D (inhibitor of macro-pinocytosis and phagocytosis, 5 μg mL^{-1}), amiloride (inhibitor of macro-pinocytosis, 100 μM), chlorpromazine (inhibitor of clathrin-mediated endocytosis, 10 μg mL^{-}

^{1}), methyl-β-cyclodextrin (inhibitor of lipid rafts/cholesterol-enriched microdomains and caveolae-mediated endocytosis, 5 mg mL^{-1}), and genistein (inhibitor of caveolae-mediated endocytosis, 200 μM), respectively, followed by treating with MCN-NPs (1 μM, calculated based on FAM labeled OVA peptide, [CpG-OVA]:[M-CP] = 1:300) for 30 min and flow cytometry analysis. The inhibition rates of uptake induced by inhibitors were calculated based on the decreased intensity of FAM fluorescence compared to the MCN-NPs treatment group. For the acridine orange staining assay,[9] the re-treated DC2.4 cells with indicated formulations (24 h) were washed three times with PBS, and further incubated with acridine orange (2.5 μg mL^{-1}) for 15 min at room temperature, then the cells were imaged under CLSM (Ex =480 nm, Em = 540 nm (green) and Ex = 561 nm, Em = 620 nm (red)) to evaluate the endo/lysosome membrane permeability of nanoparticles. For the TLR pathway analysis, peritoneal macrophages were incubated with anti-TLR1, anti-TLR2, anti-TLR4, anti-TLR5, anti-TLR6, the mixture of anti-TLR1 and anti-TLR2, and the mixture of anti-TLR2 and anti-TLR6 antibodies (20 μg mL^{-1}), at 37°C for 1 h, respectively, followed by another 1 hour of MCN-NPs (1 μM, FAM labeled OVA peptide) treatment. After fixating and sealing, cells were imaged by a confocal laser scanning microscopy.

Transcriptome Sequencing Analysis and Verification: Transcriptome sequencing analysis and RT-PCR were used to understand the regulatory mechanism of MCN-NPs. 2×10^{6} peritoneal macrophages/well were seeded in 12-well plates and stimulated by the physical mixture of OVA and CpG, CpG-OVA conjugate, M-CP, and MCN-NPs (calculated based on OVA peptide, [OVA] = 1 μM) for 24 h, respectively. mRNAs were extracted with a Universal RNA Extraction Kit (TaKaRa, Japan) following the manufacturing instructions, and some of them were sent to BGI (Shenzhen, China) for sequencing analysis based on the next-generation sequencing technology. The remaining RNA was reversely transcribed into cDNA and analyzed by real-time fluorescent quantitative PCR. In this study, genes related to the antigen processing and presentation pathways (*H2-K1*, *H2-M2*, *H2-Q7*, *H2-T22*, *Tap1*, *Tap2*, and *Tapbp*) and TLR pathways (*Tlr1*, *Tlr2*, *Tlr4*, *Tlr5*, *Tlr6*, *Tlr9*, and *MyD88*) were detected by RT-PCR.

dLNs Analysis: To visualize the *in vivo* accumulation of MCN-NPs, C57BL/6 mice were immunized with 100 μL Cy5.5-labeled CpG (15 nmol per mouse) and MCN-NPs (15 nmol Cy5.5-labeled CpG and 150 nmol M-CP per mouse) by subcutaneous vaccination at the tail base. After 24 h, the inguinal draining lymph nodes (dLNs) and other main organs were harvested and imaged by IVIS Lumina (Perkin Elmer, USA). For immunocyte typing analysis,

C57BL/6 mice (6-8 weeks) were immunized with the indicated formulations by subcutaneous injection at the tail base at days 0, 7, and 14 (n = 5). The dLNs, 7 days after the last immunization, were harvested and grounded into a single-cell suspension. Later, cells were stained with CD16/CD32 and the marker antibody of DC cells (APC-conjugated CD11c antibody), B cells (PE-conjugated CD19 antibody), or macrophages (APC-conjugated F4/80 antibody), respectively, and analyzed by a flow cytometer.

In vivo Immunization and Phenotypic Assessment of T-cells: After immunization with the indicated formulations three times, PBMCs of C57BL/6 mice were isolated by lysing the blood with RBC lysis buffer and stained with an antibody cocktail containing eFluor450-conjugated CD3e, FITC-conjugated CD8a, and APC-conjugated CD4 antibodies. For the proportion analysis of OVA-specific $CD8^+$ T-cells, the antibody cocktail containing eFluor450-conjugated CD3e, FITC-conjugated CD8a, and APC-conjugated H2kb OVA tetramer was used. To assess the CTL cytotoxicity of lymphocytes, the ELISPOT assay of IFN-γ was performed with splenocytes from immunized mice on day 21 following the manufacturer's instructions. In this test, 25 kDa branched-PEI (80 μg PEI) and PEI-NPs (nanoparticles assembled from 80 μg PEI and 15 nmol CpG-OVA conjugate) were included as a positive group as described in the previous report.[10] Re-proliferation ability of lymphocytes was also evaluated by CCK8 tests after incubating with 50 μg mL^{-1} OVA peptide for 60 h.

Immunogenicity Assessment of Peptide Carriers: The immunogenicity of M-CP was evaluated by detecting the anti-M-CP or M-LP antibody in serum as mentioned in the previous report.[11] After three times immunization, the serum at day 21 was harvested for further ELISA tests. In brief, biotinylated M-CP or M-LP (50 μM, 200 μL) was added to a neutrAvidin coated 96-well plate (Thermo Fisher, 15129) at 4°C for 12 h. The plate was washed 3 times with PBST (0.1% Tween 20-containing PBS buffer) and then incubated with 5% BSA for 1 h. After washing with PBST, mouse serum was added to the plate at a dilution of 10 for 1 h, and the plate was washed three times again. Then HRP-labeled mouse antibody (CST, 7076) was added to the plate with a 2,000× dilution for 1 h. After washing five times with PBST, substrate solution/well (90 μL, Thermo Scientific, N301) was added. 15-30 min later, the reaction was ended by adding stop solution (50 μL, CST, 7002P4), and the plate was measured by a Microplate Reader at 450 nm.

Immune Oncology Prevention: For lung metastasis immune prevention, vaccinated mice were challenged with 5×10^5 B16-OVA cells per mouse by intravenous injection at day 0. 20 days later, the lungs were excised and stained in Fekete's solution for the enumeration of lung metastatic nodules.[1a] The invasion of B16-OVA tumor cells to normal lung cells was evaluated by the H&E-stained assay. In the tumor growth inhibition model, tumor volume and weight of the vaccinated mice, which were subcutaneously challenged with 1×10^6 B16-OVA cells per mouse at day 0, were monitored at two-day intervals until the tumor volume reached 1500 mm^3 (tumor volume = $0.5\times length\times width^2$). To estimate the safety of nano-vaccines, the main organs were harvested and stained with H&E. The statistical endpoints of the survival curve were designated when the average tumor diameter exceeds 20 mm, the tumor growth exceeds 10% of the original body weight, or when the mouse dies.

Tumor Immunotherapy: For tumor immunotherapy studies, each mouse was subcutaneously injected with 1×10^6 B16-OVA cells (Murine melanoma cell with exogenous OVA protein expression) or MC-38 cells (cell line of murine carcinoma of colon without exogenous OVA protein expression) on the right flank. 6-7 days later, mice were randomly divided and injected with the different formulations at 7 days intervals. Tumor volume and body weight were monitored as described above. Specifically, in the MC-38 tumor model, the cargo of MCN-NPs vaccines was replaced by the Adpgk neoantigen, which was derived from MC-38 tumor mutation as illustrated in the previous report.[12] The tumors were harvested and further analyzed the cytotoxic T lymphocyte ($CD8^+$ T-cell) infiltration in the MC-38 tumor by the immunohistochemical (IHC) study. After imaging by scanning microscopy, the proportion of $CD8^+$ T-cells in tumor tissues was obtained based on the Aipathwell analysis system provided by Servicebio Technology (Wuhan, China).

Statistical Analysis: All statistical results were analyzed by Prism 8 (GraphPad Software). The data are presented as mean ± SEM (n = 3) from at least three independent experiments and analyzed by t-test, one-way ANOVA with Turkey multiple comparisons post-test or two-way ANOVA with Bonferroni post-test. Unless otherwise indicated, the comparison was considered statistically significant if $p<0.05$.

Supplementary Figures

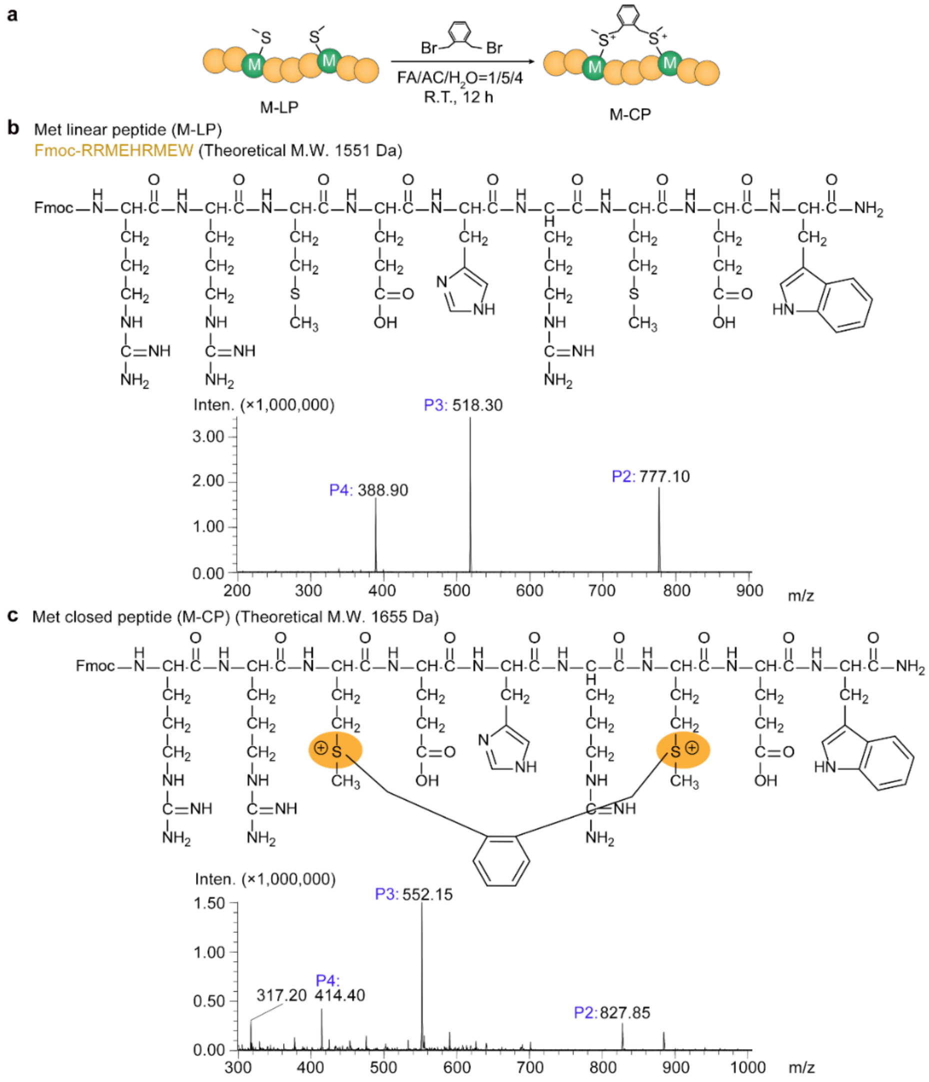

Figure S1. Preparation of sulfonium-based stapling peptide (M-CP). a) Schematic illustration of the construction of the sulfonium-based stapling peptide (Met closed peptide, M-CP). Double-sulfonium modifications and closing of M-CP at i and i+4 positions of methionine were obtained by reacting M-LP with α, α’-dibromo-o-xylene as we illustrated in the previous report. Formic acid: acetonitrile: water (FA: AC: ddH2O, V:V: V) = 1:5:4. The chemical structures and MS results of **b**) M-LP and **c**) M-CP. The parts highlighted in orange showed the ring-closing positions of M-CP.

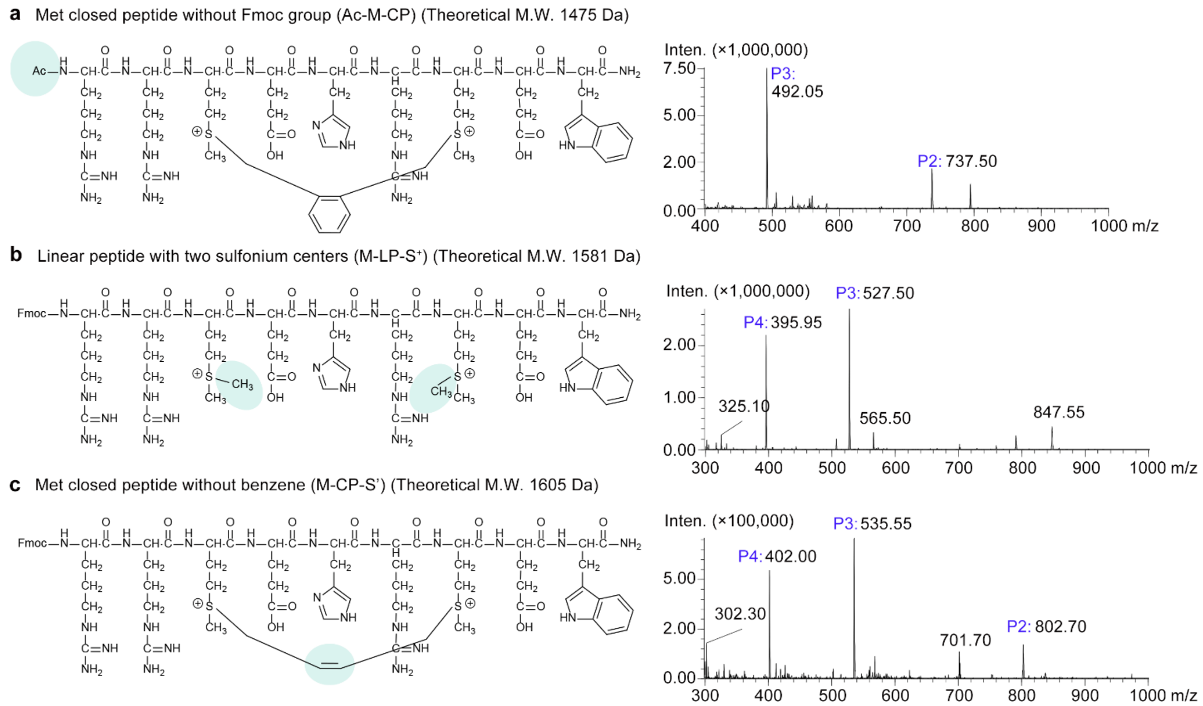

Figure S2. Preparation of redesigned peptide carriers based on M-CP. The chemical structures and MS results of **a**) Ac-M-CP, **b**) M-LP-S^+, and **c**) M-CP-S'. The parts highlighted in light green showed the change in the positions of peptides based on M-CP.

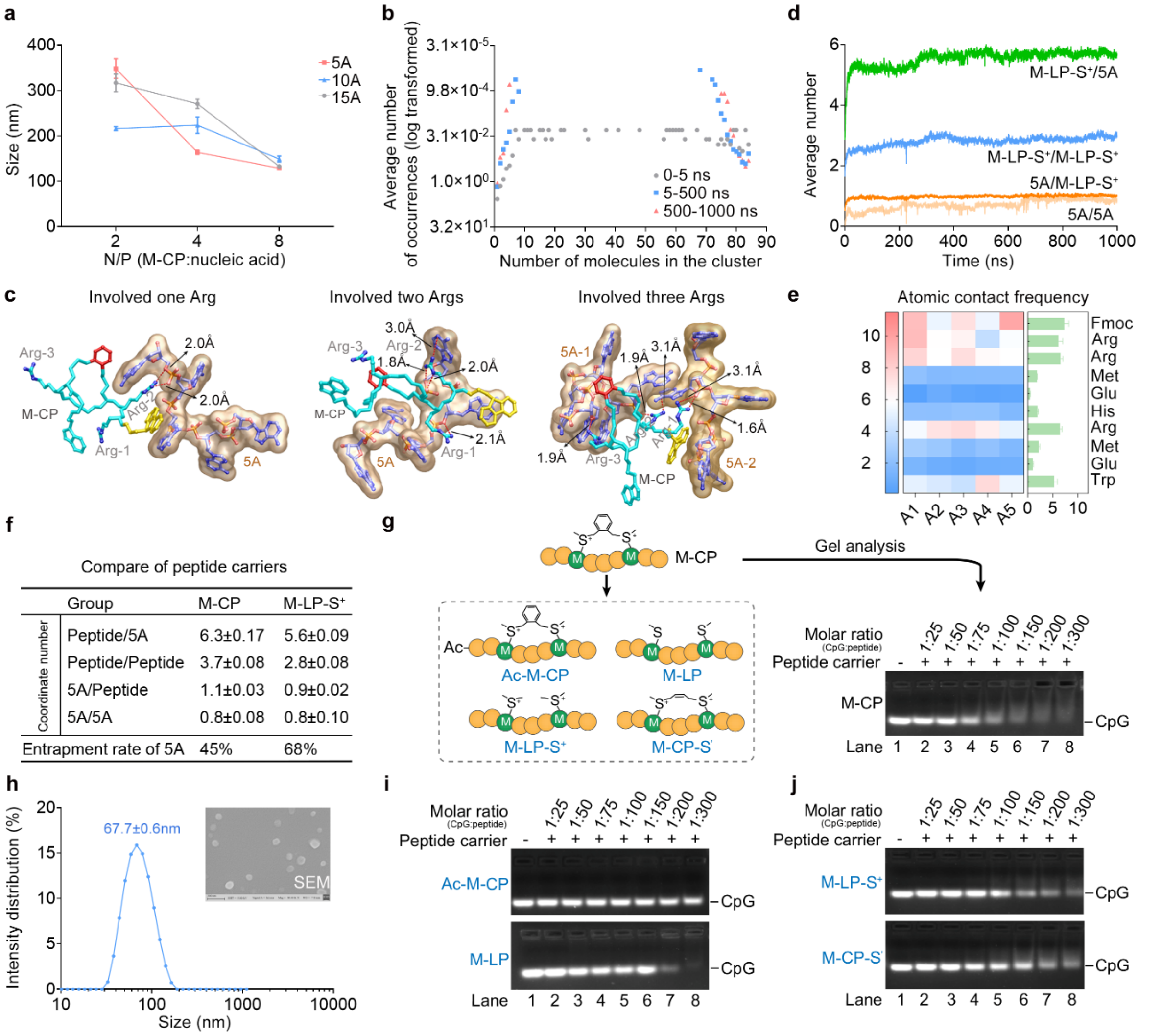

Figure S3. Analysis of the assembly behaviors of sulfonium-based stapling peptide and nucleic acid. a) Size analysis of nanoparticles assembled by M-CP with 5A (5 nt), 10A (10 nt), and 15A (15 nt) under different N/P ratios, detected by dynamic light scattering. N/P represented the ratio of the positive charge of M-CP to the number of phosphate groups in nucleic acid. **b**) The average number of occurrences of a cluster in 1000 ns in the 10×10×10 nm cubic box. 1 eq 5A and 6 eq M-CP were put into a 10×10×10 nm cubic box with real-time monitoring of cluster formation. 5 ns later, the assembled structure gradually stabilized with small (containing 0-10 molecules) and large (containing 60-90 molecules) clusters. **c**) The figures presented the potential interaction model (hydrogen bond) of arginine on M-CP with 5A. To simplify the structure, M-CP was shown as a skeleton model with Fmoc (yellow), tryptophan (light blue), closed-loop (red), and three arginines (light blue embedding with blue-green N atom). 5A was shown as the perspective surface model. Red dashed indicated the potential positions of hydrogen bond interaction. **d**) Intermolecular distribution of the nanoparticle assembled by M-LP-S$^+$ and 5A. x/y showed the average number of x molecules

around one y molecule. **e**) The heat map of the atomic contact frequencies between the amino acid residues of M-LP-S^+ and each nucleotide in 5A. The right bar chart represented the average interaction frequencies between one amino acid residue and each nucleotide residue. **f**) The assembly differences of M-CP and M-LP-S^+ with 5A, represented by the coordinate number and entrapment rate. The coordination number was expressed as the average number in 500-1000 ns. **g**) Diagram of redesigned peptide carriers based on M-CP and the gel electrophoresis (3% agarose) analysis of the assembly from M-CP and CpG ssDNA. **h**) Size and micromorphology of nanoparticles assembled with M-CP and CpG were analyzed by dynamic light scattering and SEM. [CpG]: [M-CP] = 1:200. Scale bar = 200 nm. **i,j**) Gel electrophoresis (3% agarose) analysis of the encapsulation efficiency of CpG adjuvant by different peptide carriers (Ac-M-CP, M-LP, M-LP-S^+, and M-CP-S^-).

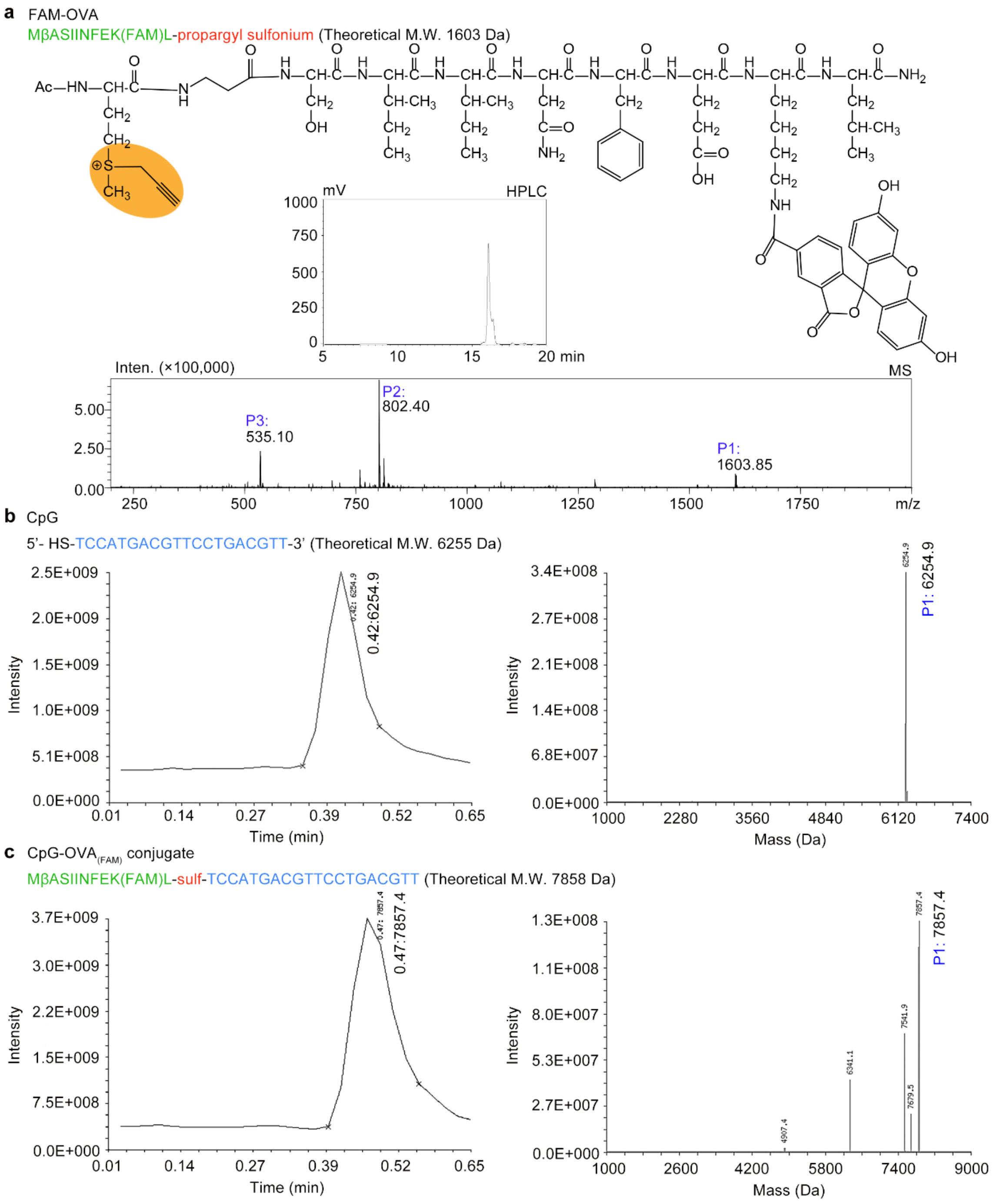

Figure S4. Preparation and characterization of CpG-OVA conjugate. a) The chemical structure and LC-MS results of OVA antigen with propargyl sulfonium. The propargyl sulfonium was introduced into the methionine of OVA peptide (FAM dye-labeled on the side chain of lysine) under acidic conditions and specifically coupled with the sulfhydryl group of CpG adjuvant under weakly alkaline conditions. **b**) The sequence and the MALDI-TOF results of sulfhydryl-modified CpG adjuvant (Theoretical molecular weight, 6255 Da). **c**) MALDI-TOF results of CpG-OVA$_{(FAM)}$ conjugate (Theoretical molecular weight, 7858 Da).

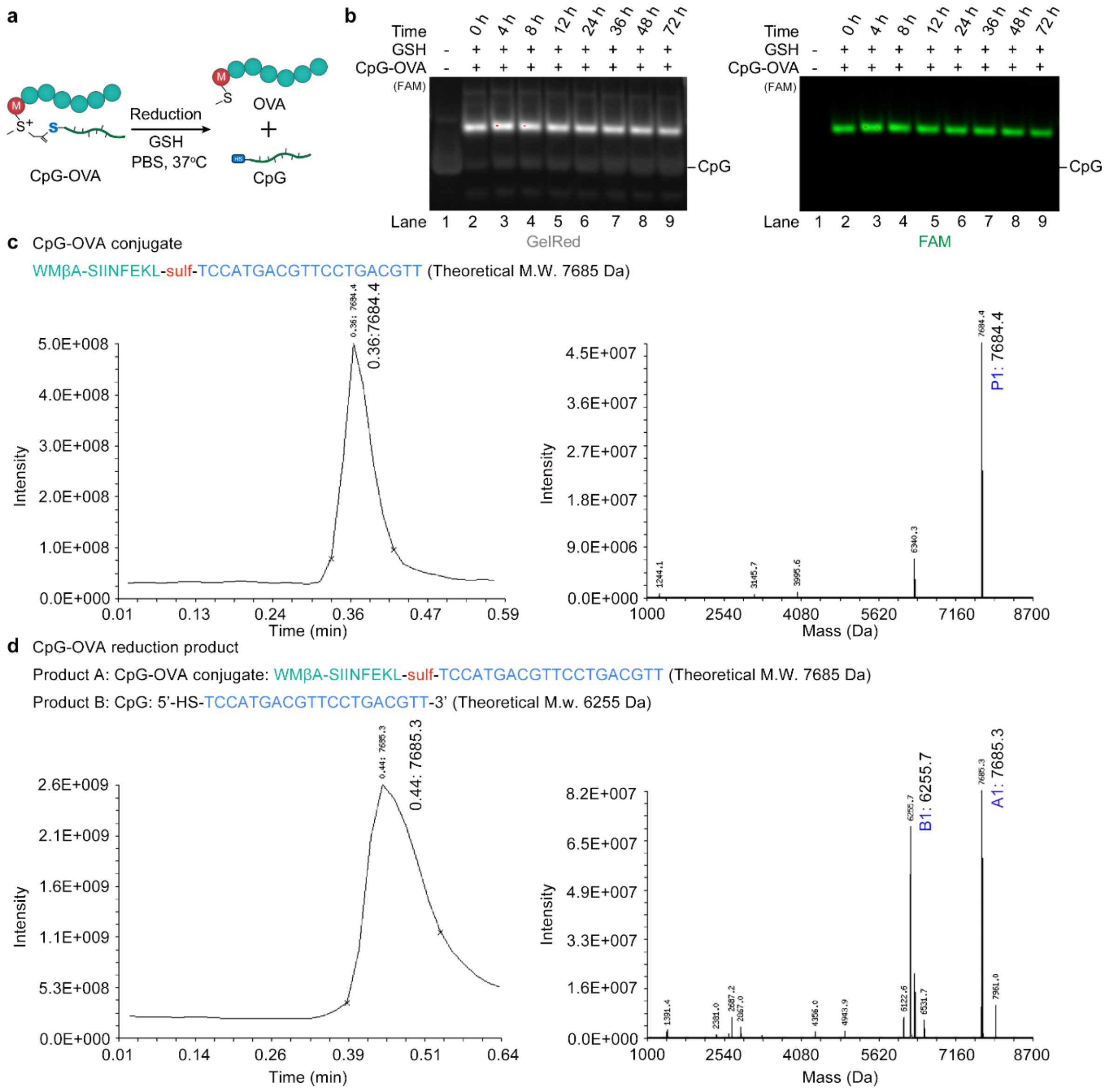

Figure S5. Reversible reduction of CpG-OVA conjugates by GSH. **a**) Schematic illustration of the reversible reduction process of the sulfonium-based conjugate by glutathione (GSH). The adjacent carbon atom of the active sulfonium center was replaced by the sulfhydryl group of GSH to release the antigen and adjuvant. **b**) Gel electrophoresis (3% PAGE) analysis of the reduction products of CpG-OVA$_{(FAM)}$ induced by GSH over time. **c**) MALDI-TOF results of CpG-OVA conjugate (Theoretical molecular weight, 7685 Da). **d**) MALDI-TOF results of the reduction products of CpG-OVA conjugate induced by GSH.

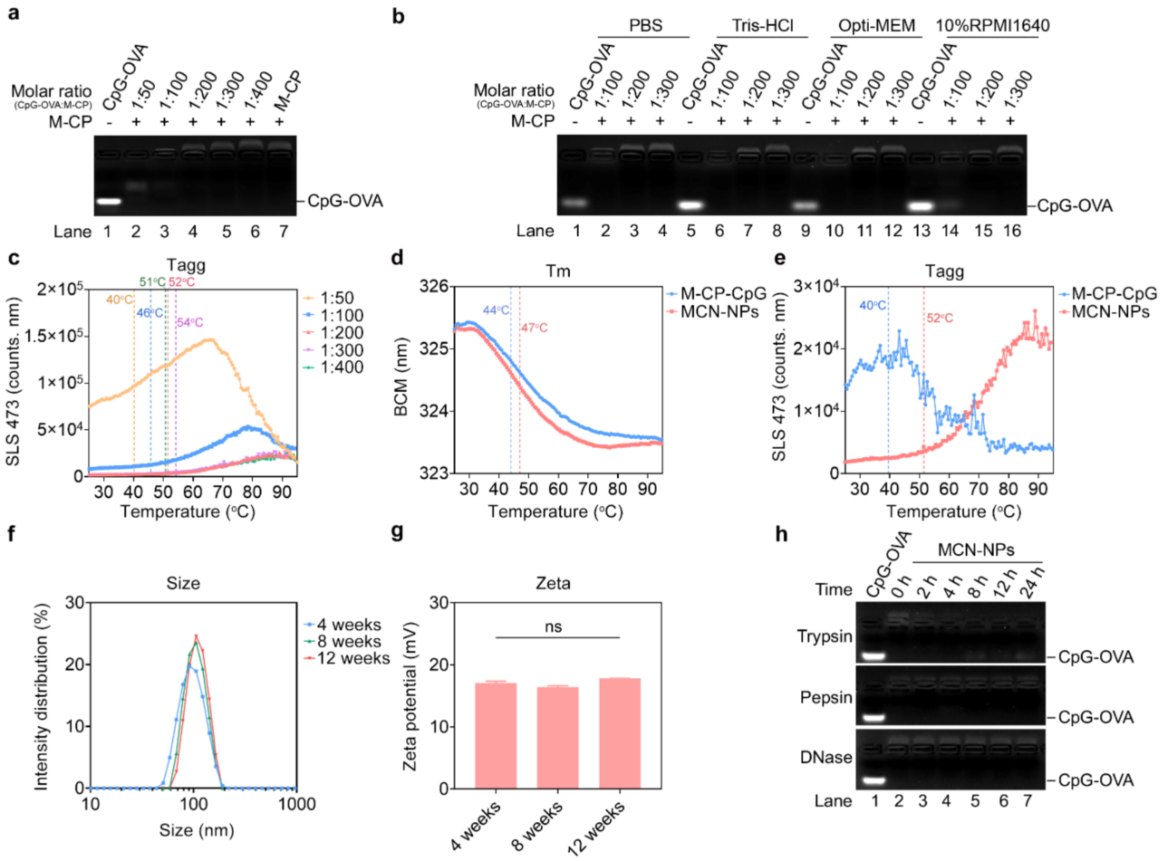

Figure S6. Characterization of MCN-NPs. Gel electrophoresis (3% agarose) analysis on the assembly of nanoparticles (MCN-NPs) of **a**) different substrate ratios and **b**) ratios under different physiological buffer conditions. **c**) Onset aggregation temperature (T_{agg}) of MCN-NPs under different substrate ratios detected by Uncle. SLS represented static light scattering analysis. The T_{agg} values of the MCN-NPs under the substrate ratios of 1:50, 1:100, 1:200, 1:300, and 1:400 ([CpG]: [M-CP]) were 40, 46, 52, 54, and 51°C, respectively. **d**) Melting temperature midpoint (T_m) of M-CP-CpG nanoparticles and MCN-NPs. The T_m values of M-CP-CpG nanoparticles and MCN-NPs were 44 and 47°C, respectively. **e**) T_{agg} of M-CP-CpG nanoparticles (40°C) and MCN-NPs (52°C) detected by Uncle. The changes of **f**) particle size and **g**) zeta potential of MCN-NPs after incubation at 4°C in 12 weeks. **h**) Gel electrophoresis (3% agarose) analysis of MCN-NPs under different physiological environments.

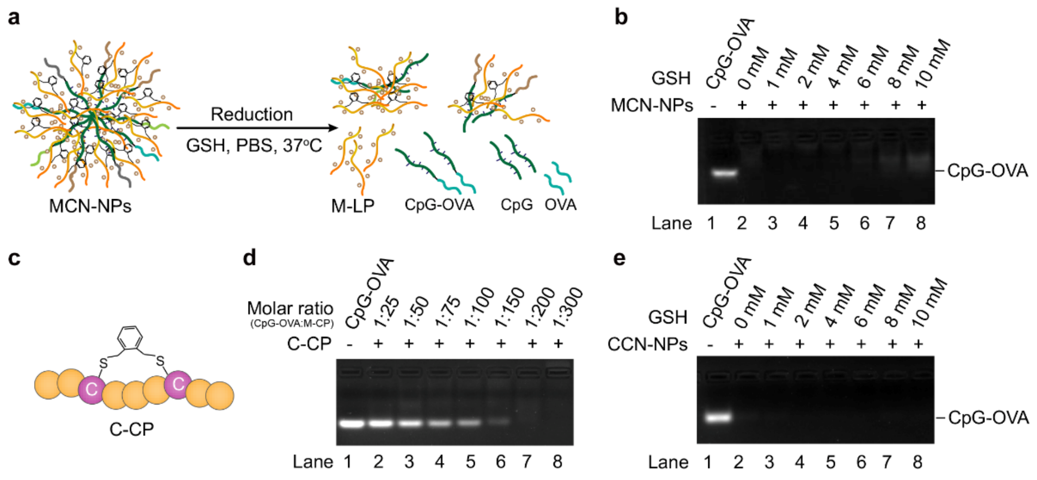

Figure S7. Reversible reduction of MCN-NPs by GSH. a) Schematic illustration of reversible disassembly of MCN-NPs induced by GSH. The active sulfonium centers of M-CP were restored by the sulfhydryl group of GSH, which would further induce carrier disintegration and internal drug release. **b**) Gel electrophoresis (3% agarose) analysis of the reduction products of MCN-NPs reduced by GSH. Nanoparticles were incubated with different concentrations of GSH in PBS buffer at 37°C for 1 h. **c**) Schematic illustration of C-CP (closed-peptide without sulfonium centers). **d**) Gel electrophoresis (3% agarose) analysis of the assembly of C-CP with CpG-OVA conjugate. **e**) Gel electrophoresis (3% agarose) analysis of the disassembled products of CCN-NPs (nanoparticles assembled by C-CP and CpG-OVA conjugate) induced by GSH. Nanoparticles were incubated with different concentrations of GSH in PBS buffer at 37°C for 1 h.

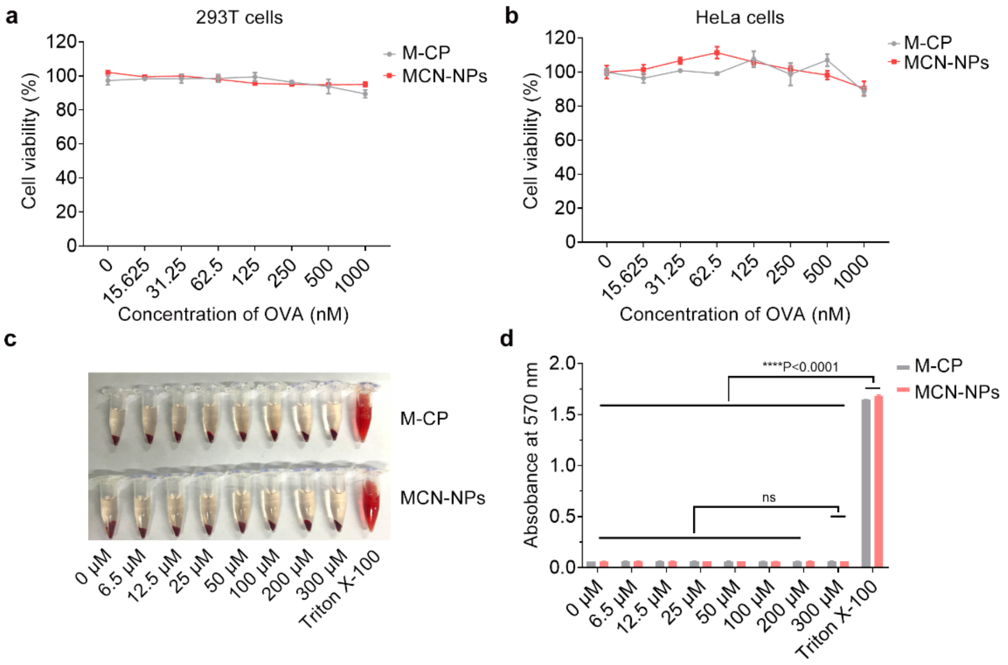

Figure S8. **Safety assessment of MCN-NPs and M-CP.** Cytotoxicity assessment of MCN-NPs and M-CP (calculated based on OVA concentration) to **a**) 293T and **b**) HeLa cells. [CpG-OVA]: [M-CP] = 1:300. Shown **c**) the image and **d**) the absorbance of the supernatants of the fresh red blood cells after incubating with MCN-NPs or M-CP (calculated based on M-CP concentration) at 37°C for 1 h. 0.1% (w/w) Triton X-100 was included as the positive control. The data were presented as mean ± SEM (n = 3) and analyzed by two-way ANOVA with Bonferroni post-test (**P*<0.05, ***P*<0.01, ****P*<0.001, *****P*<0.0001).

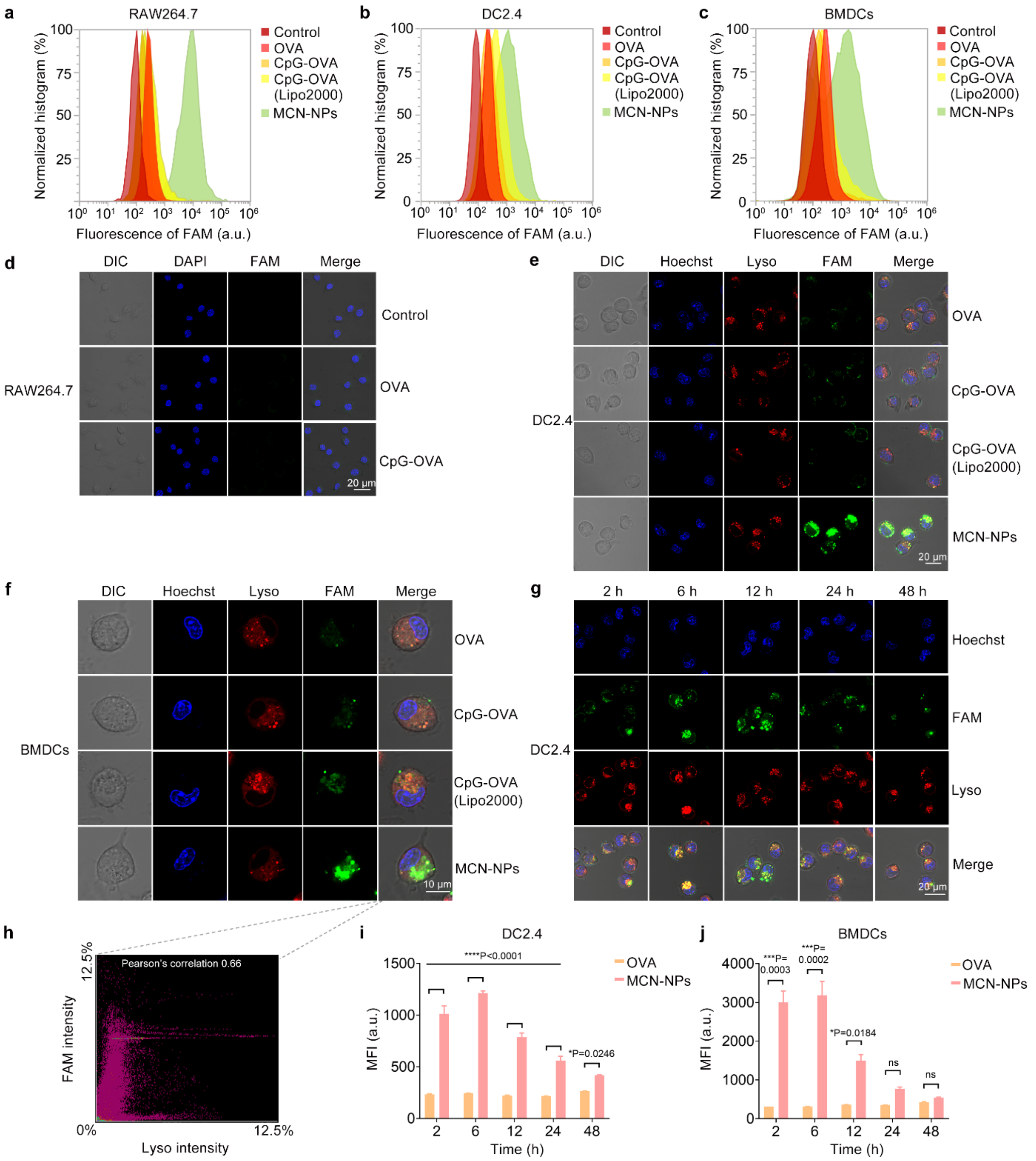

Figure S9. The uptake behaviors of MCN-NPs by immune cells. Shown the flow cytometry measurements of MCN-NPs (FAM fluorescence dye-labeled OVA peptide) incubating with **a**) RAW264.7 cells, **b**) DC2.4 cells, and **c**) BMDCs for 4 h. **d**) CLSM analysis of RAW264.7 incubating with PBS, free OVA peptide, and CpG-OVA conjugate for 4 h. DAPI (blue) was used to stain cell nucleus. OVA peptides were labeled with FAM(green). Scale bar = 20 µm. CLSM images of **e**) DC2.4 cells and **f**) BMDCs incubated with the indicated formulations (calculated based on OVA concentration) for 4 h. A commercialized lipofectamine 2000 assisted transfection group (CpG-OVA (Lipo2000)) was used as the positive control. Hoechst (blue) was used to stain the cell nucleus. FAM (green) was employed to label the OVA peptide.

Lysotracker (Lyso, red) was used as the lysosome dye. **g**) Co-localization of internalized OVA peptide and lysosomes in DC2.4 cells over time. Hoechst (blue) stained the cell nucleus. FAM (green) was used to label the OVA peptide. Lysotracker (Lyso, red) was employed as the lysosome dye. Scale bar = 20 μm. **h**) The Pearson's correlation of OVA peptide with lysosomes in the MCN-NPs treatment groups appeared in **f**). Flow cytometry measurement of the mean fluorescence intensity (MFI) of **i**) DC2.4 cells and **j**) BMDCs after incubating with OVA or MCN-NPs for 2, 6, 12, 24, and 48 h. The data were presented as mean ± SEM (n = 3) from three independent experiments. Data were analyzed by two-way ANOVA with Bonferroni post-test (*P<0.05, **P<0.01, ***P<0.001, ****P<0.0001).

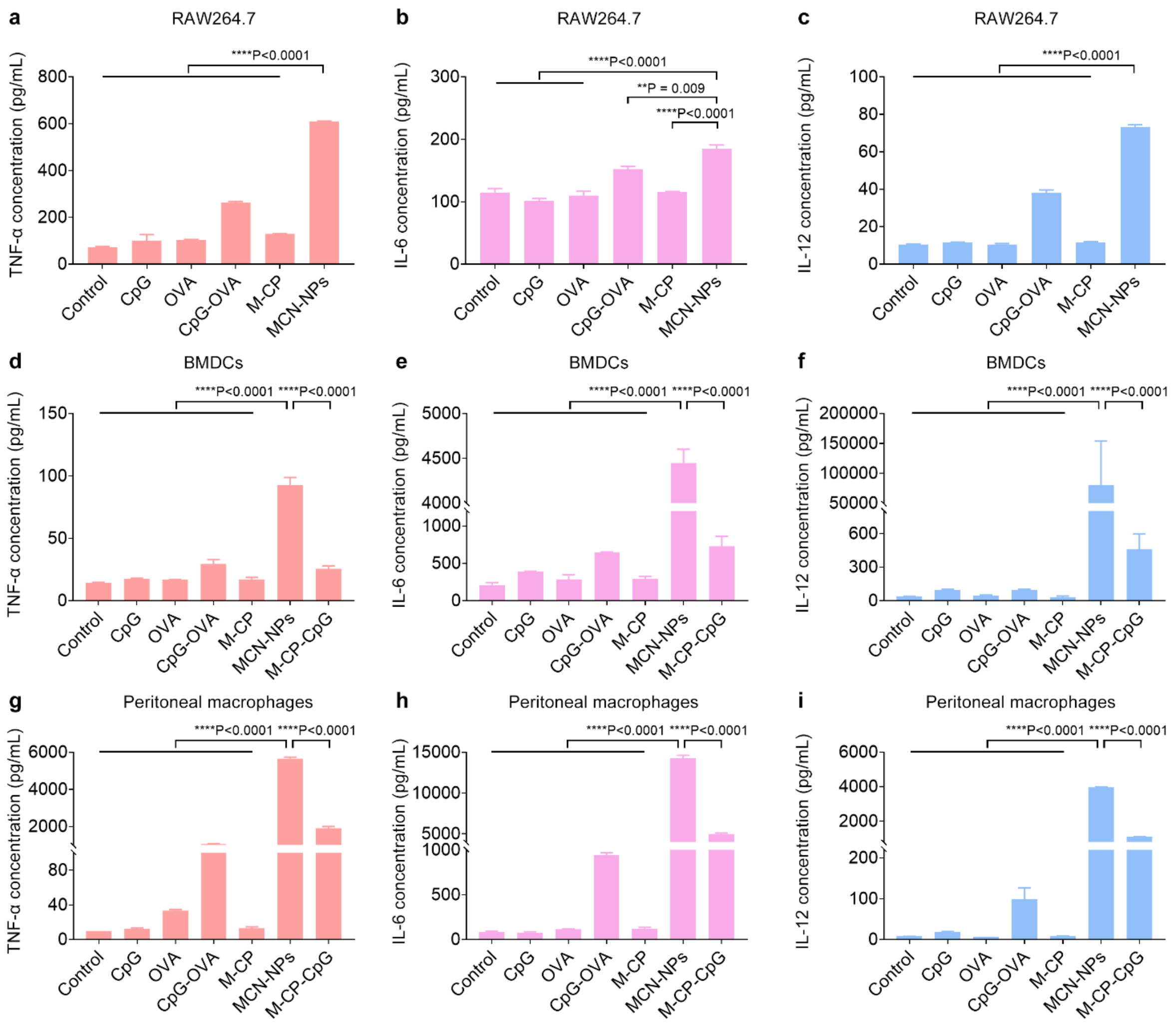

Figure S10. MCN-NPs stimulated strong cytokines secretion. The concentration of **a**) TNF-α, **b**) IL-6, and **c**) IL-12 in RAW264.7 cells culture supernatant after incubating the cells with the indicated formulations for 24 h. Concentration of **d**) TNF-α, **e**) IL-6, **f**) and IL-12 in BMDCs culture supernatant after incubating the cells with different formulations for 48 h. Concentration of **g**) TNF-α, **h**) IL-6, and **i**) IL-12 in the culture supernatant of peritoneal macrophages under different formulations for 24 h. The data were presented as mean ± SEM (n = 3) from three independent experiments. Data were analyzed by one-way ANOVA with Turkey multiple comparisons post-test (**P*<0.05, ***P*<0.01, ****P*<0.001, *****P*<0.0001).

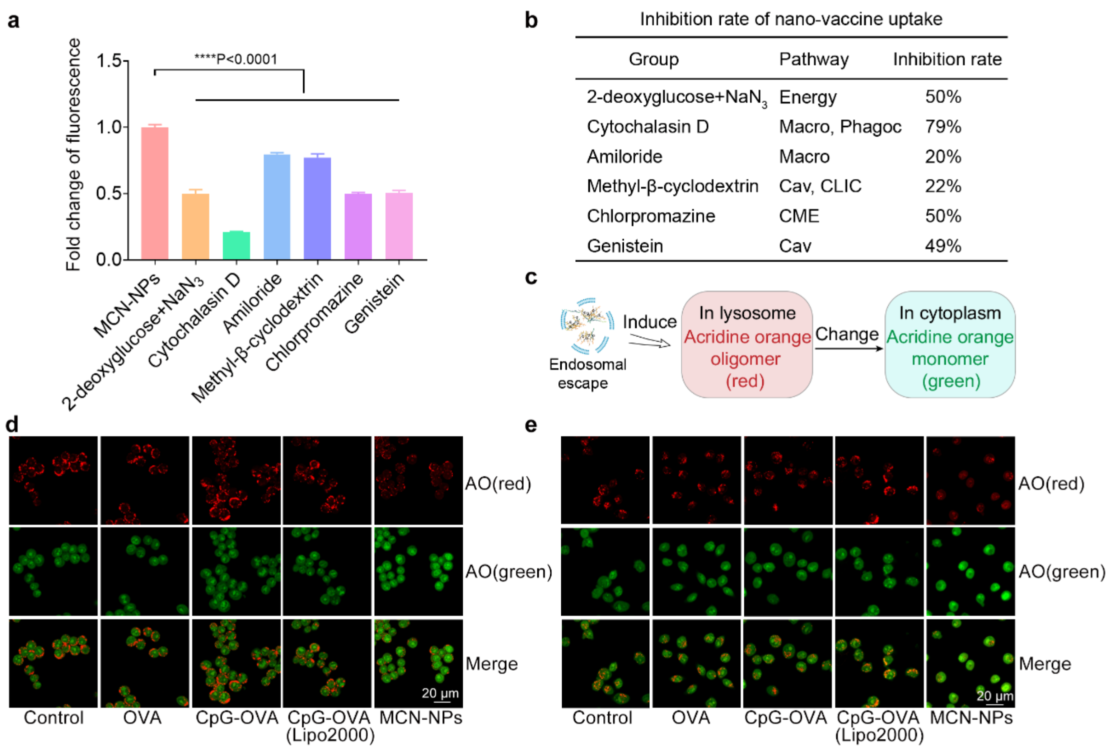

Figure S11. **The process of membrane permeation and lysosomal escape of MCN-NPs.** **a**) Fold change of DC2.4 cells' fluorescence after 30 min pre-incubation with different pathway inhibitors and 1 h treatment of MCN-NPs. Data were analyzed by one-way ANOVA with Turkey multiple comparisons post-test (****P<0.0001).**b**) Inhibition rate of MCN-NP uptake by inhibitors corresponding to **a**). (Energy: all energy-dependent pathways. Macro: micropinocytosis; Phagoc: phagocytosis micropinocytosis; Cav: caveolae-mediated endocytosis; CLIC: lipid rafts/cholesterol-enriched microdomains/caveolae; CME: clathrin-mediated endocytosis). **c**) Schematic illustration of the lysosome escape of MCN-NPs indicated by the fluorescent color change of acridine orange (AO). Once the lysosome membrane was destabilized or de-integrated, the oligomeric acridine orange in the lysosome was free to the cytoplasm and would convert into a monomer, represented by the fluorescence change from red to green. CLSM images of **d**) RAW264.7 and **e**) DC2.4 cells after 24 h incubation with the indicated formulations (calculated based on OVA concentration) and stained with acridine orange (2.5 μg mL^{-1}) for 15 min. Scale bar = 20 μm.

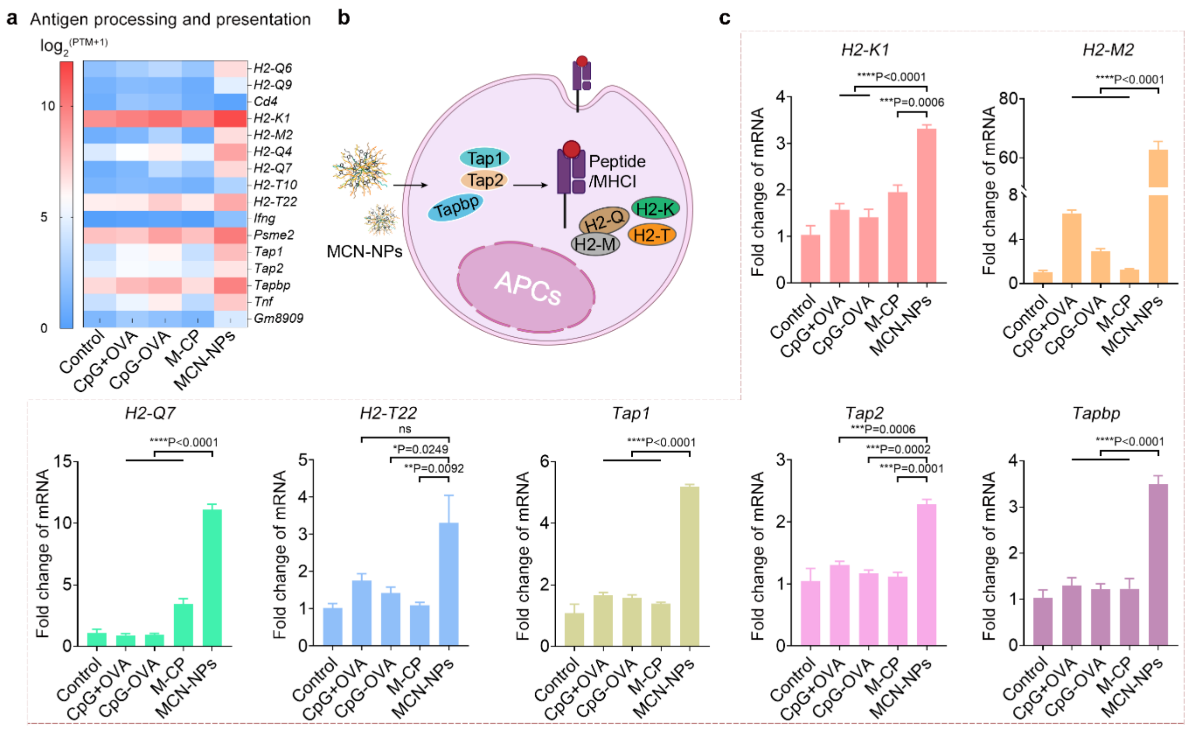

Figure S12. **MCN-NPs activate the antigen processing and presentation pathway. a**) Clustering thermogram of differential genes in antigen processing and presentation pathway after 24-h incubation with MCN-NPs. **b**) Schematic illustration of the possible antigen-presenting processes in APCs induced by MCN-NPs. **c**) The mRNA fold change of the representative differential genes related to antigen processing and presentation pathway after MCN-NPs treatment. The data were presented as mean ± SEM (n = 3) from three independent experiments. Data were analyzed by one-way ANOVA with Turkey multiple comparisons post-test (**P*<0.05, ***P*<0.01, ****P*<0.001, *****P*<0.0001).

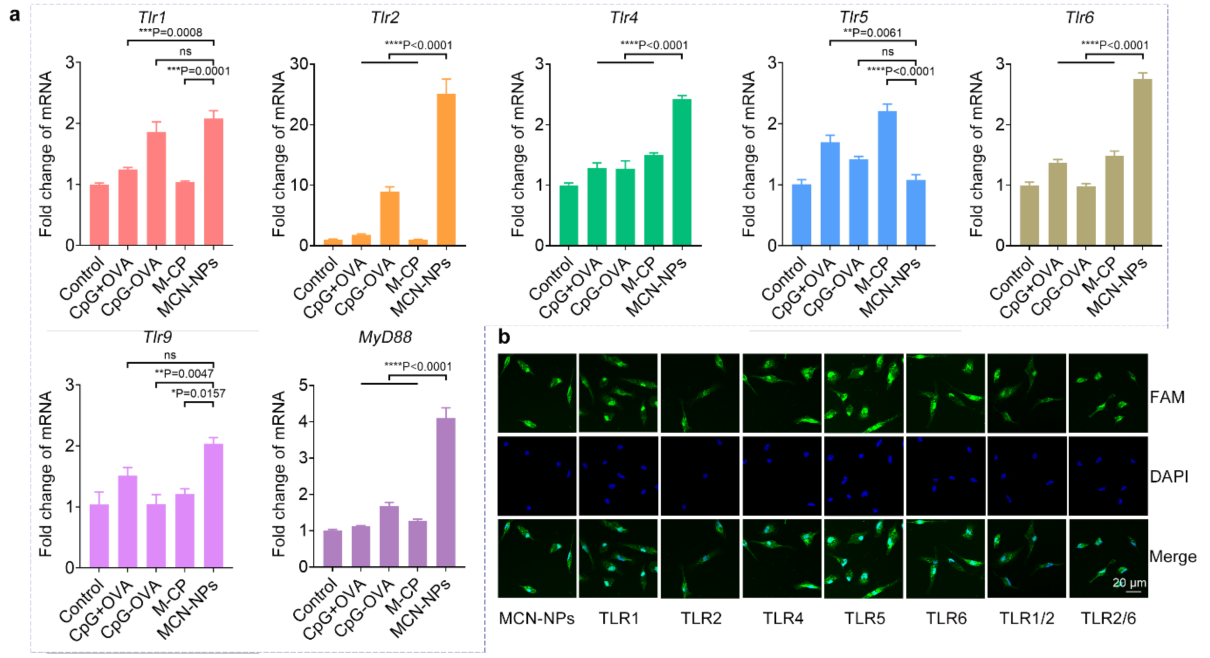

Figure S13. **MCN-NPs activate the toll-like receptor (TLR) signaling pathway. a**) The mRNA fold change of the representative differential genes related to the TLR pathway, following 24-h incubation with MCN-NPs. **b**) CLSM analysis demonstrated the MCN-NPs intake after pre-treating with anti-toll-like receptor antibodies. anti-TLR1, TLR2, TLR4, TLR5, and TLR6 antibody (20 μg mL^{-1}) was pre-incubated with peritoneal macrophages for 1 h to block the corresponding toll-like receptors on the cell membrane. DAPI (blue) stained for cell nuclei. FAM (green) was used to label the OVA peptide. Scale bar = 20 μm. The data were presented as mean ± SEM (n = 3) from three independent experiments. Data were analyzed by one-way ANOVA with Turkey multiple comparisons post-test (*P<0.05, **P<0.01, ***P<0.001, ****P<0.0001).

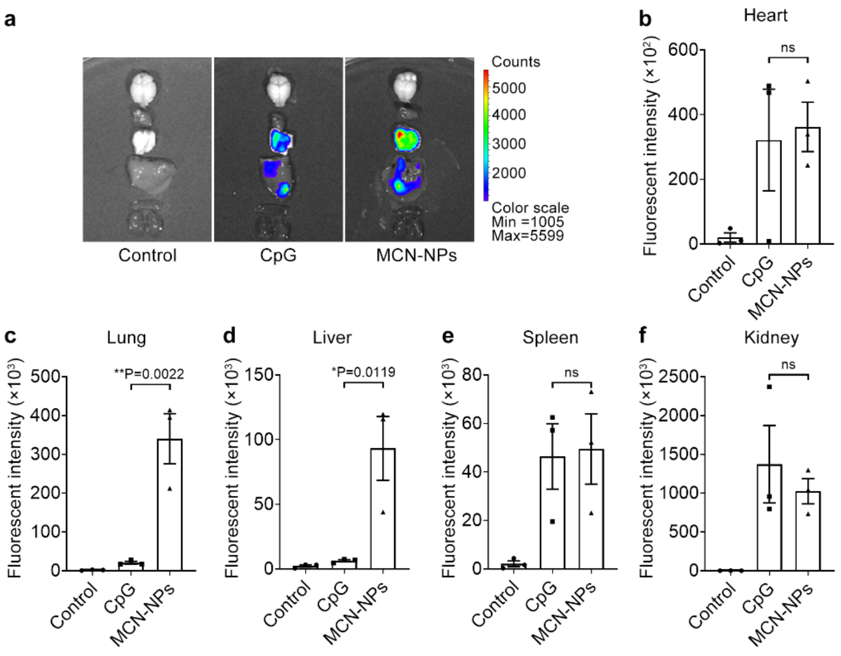

Figure S14. **The metabolize behaviors of MCN-NPs in the main organs of mice. a)** Fluorescence signals in the main organs were quantified with IVIS 24 hours after subcutaneous injection with PBS, 15 nmol free CpG, or MCN-NPs (Cy5.5 labeled CpG) at the tail base of C57BL/6 mice. Intensity statistics in **b**) the heart, **c**) lung, **d**) liver, **e**) spleen, and **f**) kidney. Data were analyzed by one-way ANOVA with Turkey multiple comparisons post-test (**P*<0.05, ***P*<0.01, ****P*<0.001, *****P*<0.0001).

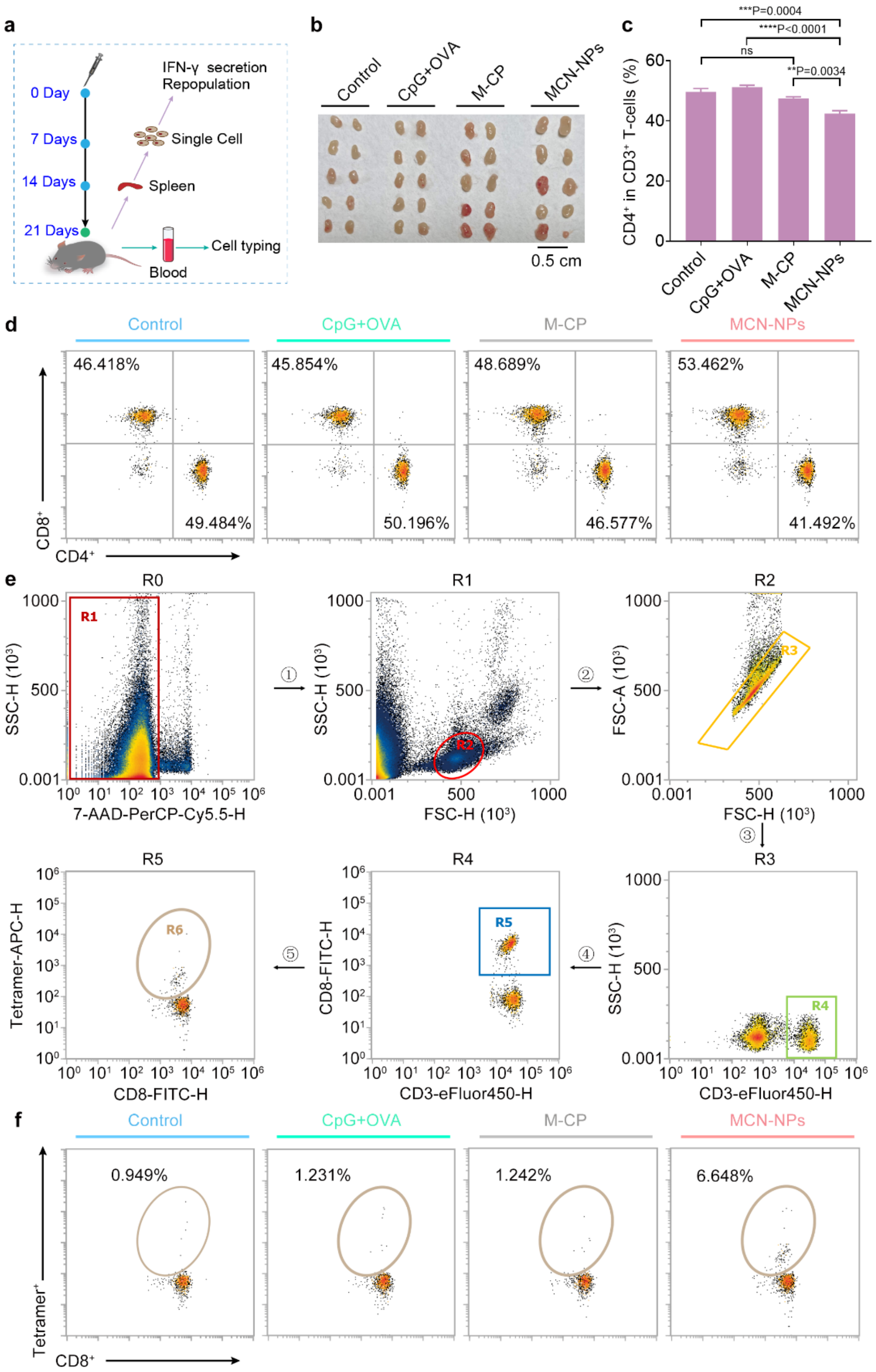

Figure S15. MCN-NPs induce the activation of the immune system *in vivo*. a) Schematic illustration showing the immunization plan and immunocyte analysis in the spleen and blood after three immunizations. **b**) Picture of the inguinal draining lymph nodes at day 7 post-immunization after immunizing three times with the indicated formulations (15 nmol CpG and 15 nmol OVA peptide) (n = 5). Scale bar = 0.5 cm. **c**) The percentage of $CD4^+$ T-cells in $CD3^+$ T-cells at day 21. **d**) The representative scatter plots of $CD4^+$ T-cells and $CD8^+$ T-cells in

peripheral blood at day 21. **e**) Gating strategy to evaluate the percentage of SIINFEKL-specific $CD8^+$ T-cells in $CD3^+$ T-cells ($Tetramer^+CD8^+CD3^+$ T-cells) at day 21. **f**) The representative scatter plots of SIINFEKL-specific $CD8^+$ T-cells in peripheral blood at day 1 post-immunization after three immunizations. The data were presented as mean ± SEM (n = 3) from three independent experiments. Data were analyzed by one-way ANOVA with Turkey multiple comparisons post-test (*P<0.05, **P<0.01, ***P<0.001, ****P<0.0001).

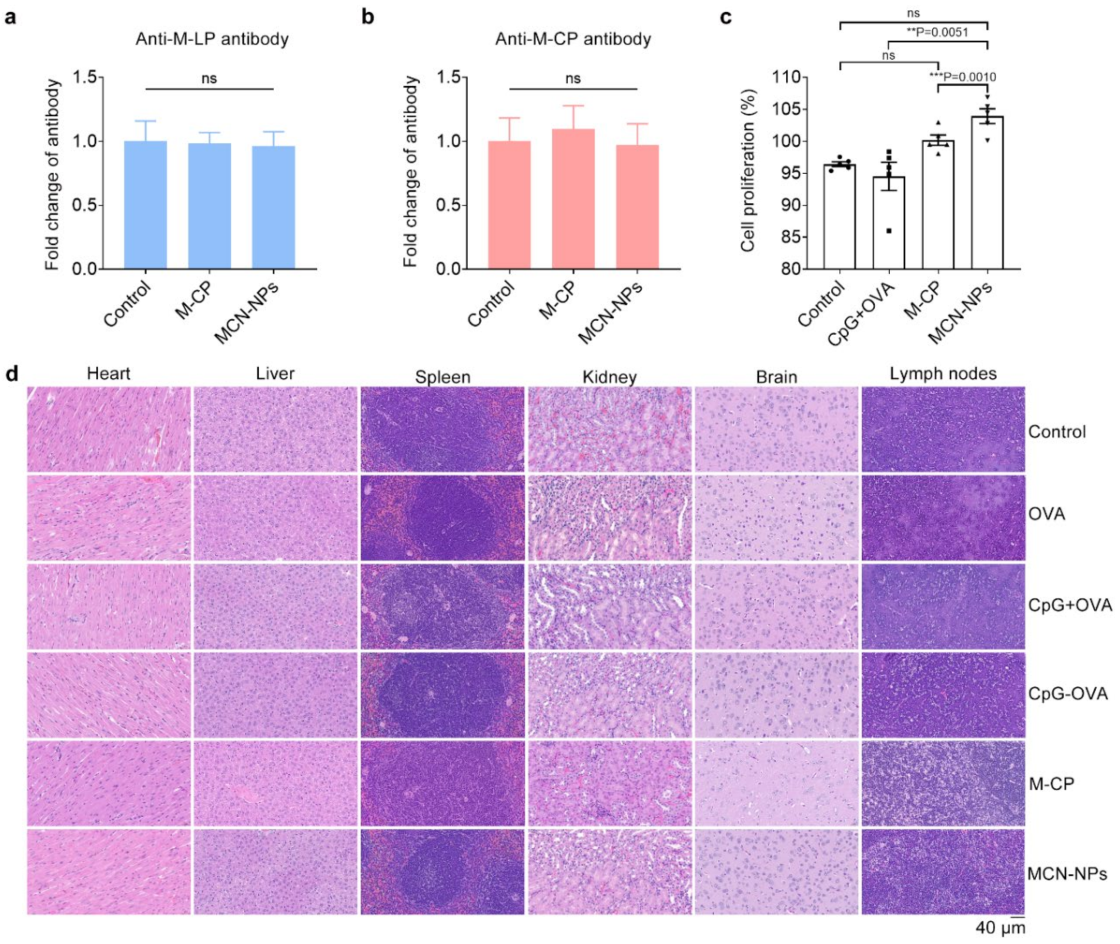

Figure S16. **MCN-NPs show good safety *in vivo*.** Immunogenicity analysis of the M-CP nano-carriers indicated by the detection of **a**) the anti-M-LP or **b**) anti-M-CP antibodies in the M-CP and MCN-NPs treatment groups. Serums of mice at day 7 post-immunization after three immunizations with M-CP (150 nmol M-CP) or MCN-NPs (15 nmol CpG-OVA and 150 nmol M-CP) (n = 3) were analyzed by ELISA. **c**) Repopulation rate of immunocytes in the spleen after re-stimulation with OVA peptide at day 7 post-immunization. The data were presented as mean ± SEM (n = 3) from three independent experiments. Data were analyzed by one-way ANOVA with Turkey multiple comparisons post-test (*P<0.05, **P<0.01, ***P<0.001, ****P<0.0001). **d**) H&E-staining of main organs collected from the lung metastasis mice model at day 20. Scale bar = 40 μm.

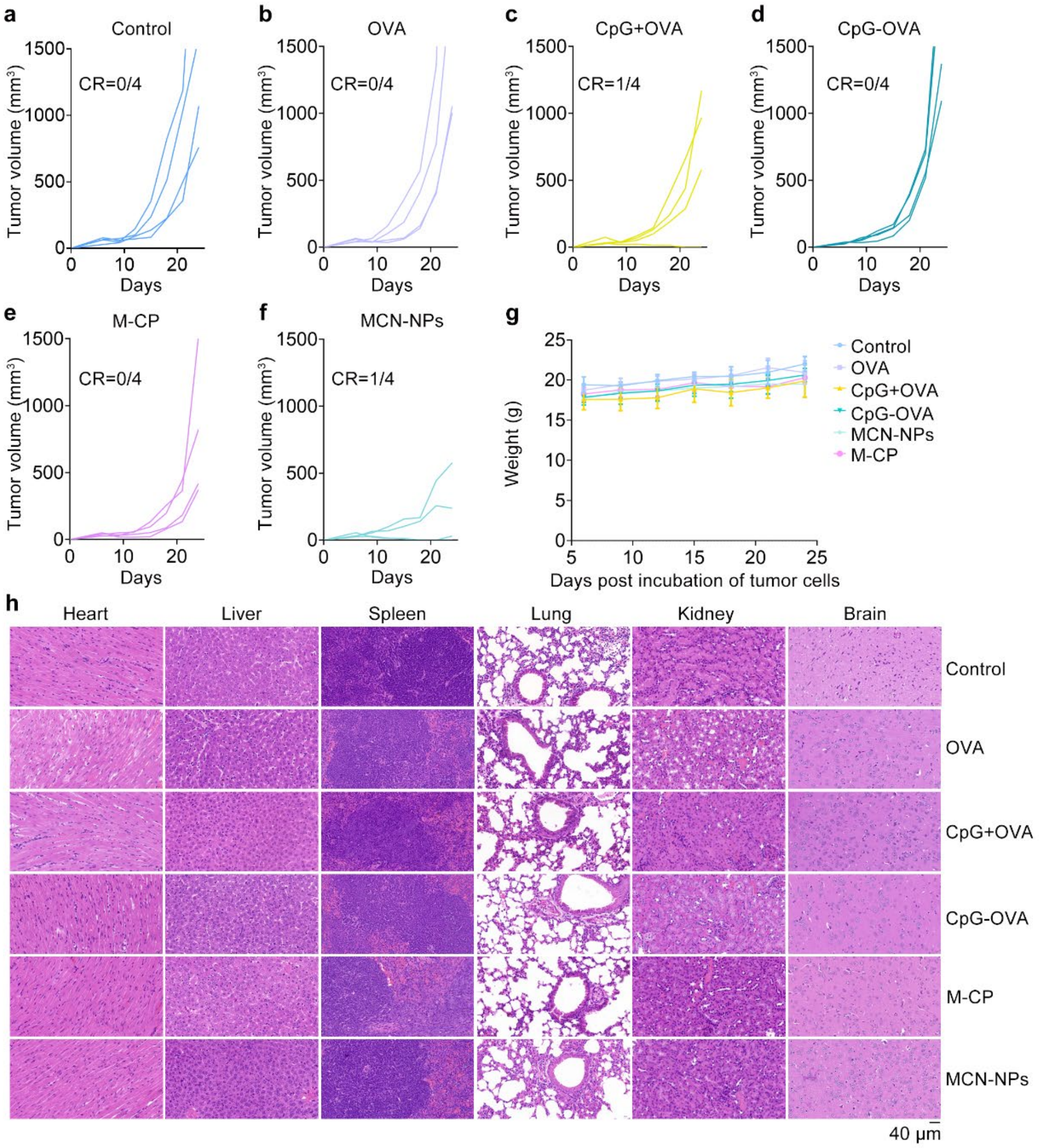

Figure S17. **MCN-NPs strongly inhibit B16-OVA tumor growth. a-f**) The individual tumor growth curves of the B16-OVA tumor (n = 4). C57BL/6 mice were subcutaneously injected with 1×10^6 B16-OVA cells at day 0, vaccinated with the indicated formulations (15 nmol CpG and 15 nmol OVA antigen) at days 6, 13, and 20, and the tumor growth was recorded over time. CR, tumor complete response rate. **g**) The body weight growth curves of B16-OVA tumor-bearing mice. **h**) H&E-stained main organ sections collected from the B16-OVA tumor-bearing mice at day 24. Scale bar = 40 μm.

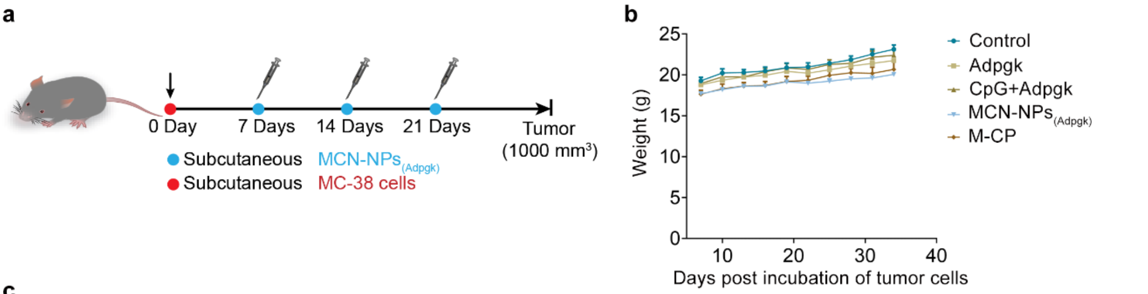

c Proportion of $CD8^+$ T-cells in tumor tissue

Group	Positive cells ($CD8^+$/Total cells,%)[a]	Positive cells density ($number/mm^2$)[b]	H-Score[c]
Control	1.86	258	3.97
Adpgk	1.74	252	3.79
CpG+Adpgk	0.89	131	1.90
M-CP	2.49	375	5.53
MCN-NPs(Adpgk)	4.94	706	11.38

[a]Positive cells = Number of positive cells/total cells number;
[b]Positive cells density = Number of positive cells/the tested area of tissue;
[c]H-Score = $\sum$（pi×i）=（percentage of weak intensity ×1)+(percentage of moderate intensity ×2)+(percentage of strong intensity ×3)

Figure S18. **MCN-NPs(Adpgk) for MC-38 tumor immunotherapy. a**) Schematic illustration of the MCN-NPs(Adpgk) administration plan for personalized tumor therapy. C57BL/6 mice were subcutaneously injected with 1×10^6 MC-38 cells at day 0, and vaccinated with the indicated formulations (15 nmol CpG and 15 nmol Adpgk neoantigen) at days 7, 14, and 21 at the tail base. The changes in tumor weight were recorded until the tumor volume reached 1000 mm^3 (n = 5). **b**) The body weight growth curves of MC-38 tumor-bearing mice. **c**) Proportion of $CD8^+$ T-cells in MC-38 tumor showing the infiltration of immunocytes in the tumor tissues after nano-vaccine (MCN-NPs(Adpgk)) treatments.

Supplementary Tables

Table S1. The ssDNAs were used to evaluate the assembly performance of peptide carriers.

Name of ssDNA	sequence (5'→3')	Length (nt)
5A	AAAAA	5
10A	AAAAAAAAAA	10
15A	AAAAAAAAAAAAAAA	15
10T	TTTTTTTTTT	10
20-scramble	GTCTGACCTGACTTGTTCCA	20
30-scramble	ACATTCCTAAGTCTGAAACATTACAGCTTG	30
CpG[a)]	TCCATGACGTTCCTGACGTT	20

[a)] CpG without modification was used to evaluate the assembly performance of peptide carriers. For the preparation of CpG-neoantigen conjugates, the 5'-end of CpG was modified with sulfhydryl and separated with six carbon atoms. Azide-modified CpG was purchased from Invitrogen (Shanghai, China) for the preparation of CpG-OVA$_{(click)}$.

Table S2. Primers for RT-PCR analysis.

Name of ssDNA	sequence (5'→3')[b)]	Length (nt)
H2-K1-F	ACCAGCAGTACGCCTACGA	19
H2-K1-R	AACCAGAACAGCAACGGTCG	20
H2-M2-F	CCCACTCCTTGCGGTACTTC	20
H2-M2-R	GCCTCATTGTCAAAATGCACAAA	23
H2-T22-F	TCCCTTTGGGTTCACACTCG	20
H2-T22-R	AGTCGTCCATGCTCTTGTTGT	21
Tap1-F	GGACTTGCCTTGTTCCGAGAG	21
Tap1-R	GCTGCCACATAACTGATAGCGA	22
Tap2-F	CTGGCGGACATGGCTTTACTT	21
Tap2-R	CTCCCACTTTTAGCAGTCCCC	21
Tapbp-F	AAGCAGCATGGAGTTCACTATG	22
Tapbp-R	TAGCACCTTGAGGAGTCCGAG	21
Tlr1-F	TTTGTCCCACAATGAGCTAAAGG	23
Tlr1-R	TTCTTTGCATATAGGCAGGGC	21
Tlr2-F	TCTAAAGTCGATCCGCGACAT	21
Tlr2-R	CTACGGGCAGTGGTGAAAACT	21
Tlr4-F	GCCTTTCAGGGAATTAAGCTCC	22
Tlr4-R	GATCAACCGATGGACGTGTAAA	22
Tlr5-F	TGGGGACCCAGTATGCTAACT	21
Tlr5-R	CCACAGGAAAACAGCCGAAGT	21
Tlr6-F	AGCCAAGACAGAAAACCCATC	21
Tlr6-R	GGGGTCATGCTTCCGACTAT	20
Tlr9-F	ACGGGAACTGCTACTACAAGA	21
Tlr9-R	CCCAGCTTGACAATGAGGTTAT	22
MyD88-F	CACCTGTGTCTGGTCCATTG	20
MyD88-R	AGGCTGAGTGCAAACTTGGT	20
GAPDH-F	TGACCTCAACTACATGGTCTACA	23
GAPDH-R	CTTCCCATTCTCGGCCTTG	19

[b)] All primers corresponded to mouse genes. F: forward primer. R: reverse primer.

Supplementary References

[1] a) Y. P. Zhang, H. K. Xu, L. Y. Jiang, Z. D. Liu, C. S. Lian, X. F. Ding, C. Wan, N. Liu, Y. A. Wang, Z. Q. Yu, L. Z. Zhu, F. Yin, Z. G. Li, *Acs Nano* **2022**, 16, 19509; b) S. L. Liu, Q. Jiang, X. Zhao, R. F. Zhao, Y. N. Wang, Y. M. Wang, J. B. Liu, Y. X. Shang, S. Zhao, T. T. Wu, Y. L. Zhang, G. J. Nie, B. Q. Ding, *Nat. Mater.* **2021**, 20, 421.

[2] I. R. Sasselli, R. V. Ulijn, T. Tuttle, *Phys. Chem. Chem. Phys.* **2016**, 18, 4659.

[3] J. Huang, S. Rauscher, G. Nawrocki, T. Ran, M. Feig, B. L. de Groot, H. Grubmuller, A. D. MacKerell, *Nat. Methods* **2017**, 14, 71.

[4] W. B. Yu, X. B. He, K. Vanommeslaeghe, A. D. MacKerell, *J. Comput. Chem.* **2012**, 33, 2451.

[5] M. J. Abraham, T. Murtola, R. Schulz, S. Páll, J. C. Smith, B. Hess, E. Lindahl, *SoftwareX* **2015**, 1-2, 19.

[6] Y. W. Kim, T. N. Grossmann, G. L. Verdine, *Nat. Protoc.* **2011**, 6, 761.

[7] Z. F. Hou, D. Y. Wang, Y. Li, R. T. Zhao, C. Wan, Y. Ma, C. S. Lian, F. Yin, Z. G. Li, *J. Org. Chem.* **2020**, 85, 1698.

[8] N. Gong, Y. Zhang, *Nat. Nanotechnol.* **2020**, 15, 1053.

[9] J. Xu, J. Lv, Q. Zhuang, Z. J. Yang, Z. Q. Cao, L. G. Xu, P. Pei, C. Y. Wang, H. F. Wu, Z. L. Dong, Y. Chao, C. Wang, K. Yang, R. Peng, Y. Y. Cheng, Z. Liu, *Nat. Nanotechnol.* **2020**, 15, 1043.

[10] J. R. Cubillos-Ruiz, X. Engle, U. K. Scarlett, D. Martinez, A. Barber, R. Elgueta, L. Wang, Y. Nesbeth, Y. Durant, A. T. Gewirtz, C. L. Sentman, R. Kedl, J. R. Conejo-Garcia, *J. Clin. Invest.* **2009**, 119, 2231.

[11] J. Wang, Y. Liu, Y. J. Liu, S. Q. Zheng, X. Wang, J. Y. Zhao, F. Yang, G. Zhang, C. Wang, P. R. Chen, *Nature* **2019**, 569, 509.

[12] R. Kuai, L. J. Ochyl, K. S. Bahjat, A. Schwendeman, J. J. Moon, *Nat. Mater.* **2017**, 16, 489.
